# A numerical compass for experiment design in chemical kinetics and molecular property estimation

**DOI:** 10.1186/s13321-024-00825-0

**Published:** 2024-03-22

**Authors:** Matteo Krüger, Ashmi Mishra, Peter Spichtinger, Ulrich Pöschl, Thomas Berkemeier

**Affiliations:** 1 Multiphase Chemistry Department, Max Planck Institute for Chemistry, Hahn-Meitner-Weg 1, Mainz, 55128 Rhineland Palatinate Germany; 2 Institute for Atmospheric Physics, Johannes Gutenberg University, Johann-Joachim-Becher-Weg 21, Mainz, 55128 Rhineland Palatinate Germany

**Keywords:** Chemical kinetics, QSAR, Design of experiments (DOE), Global optimization, Inverse problem, Ensemble methods, Multiphase chemistry, Machine learning

## Abstract

Kinetic process models are widely applied in science and engineering, including atmospheric, physiological and technical chemistry, reactor design, or process optimization. These models rely on numerous kinetic parameters such as reaction rate, diffusion or partitioning coefficients. Determining these properties by experiments can be challenging, especially for multiphase systems, and researchers often face the task of intuitively selecting experimental conditions to obtain insightful results. We developed a numerical compass (NC) method that integrates computational models, global optimization, ensemble methods, and machine learning to identify experimental conditions with the greatest potential to constrain model parameters. The approach is based on the quantification of model output variance in an ensemble of solutions that agree with experimental data. The utility of the NC method is demonstrated for the parameters of a multi-layer model describing the heterogeneous ozonolysis of oleic acid aerosols. We show how neural network surrogate models of the multiphase chemical reaction system can be used to accelerate the application of the NC for a comprehensive mapping and analysis of experimental conditions. The NC can also be applied for uncertainty quantification of quantitative structure–activity relationship (QSAR) models. We show that the uncertainty calculated for molecules that are used to extend training data correlates with the reduction of QSAR model error. The code is openly available as the Julia package *KineticCompass*.

**Graphical Abstract:**

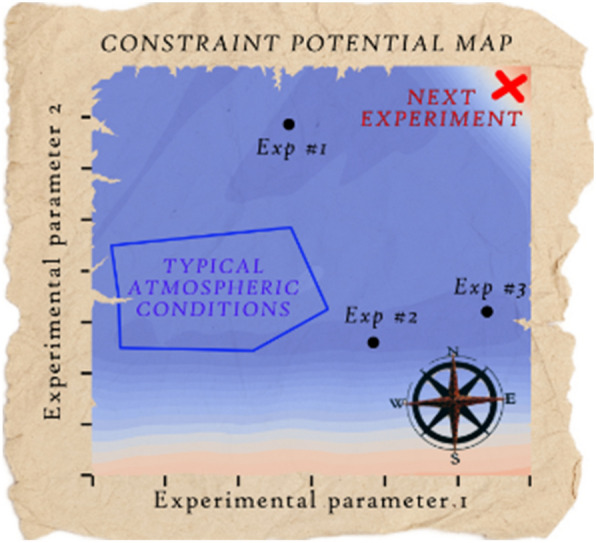

**Supplementary Information:**

The online version contains supplementary material available at 10.1186/s13321-024-00825-0.

## Introduction

In multiphase chemical kinetics, the rate of change in complex systems can be described by resolving mass transport and chemical reactions at the molecular process level [1, 2]. While the underlying physical and chemical principles are well understood, the individual processes are inherently coupled and the chemical and physical parameters, such as reaction, diffusion, or partitioning coefficients, are often unknown or poorly constrained [3, 4]. The integration of these processes occurring in parallel or in sequence often requires computational kinetic models (KM). KM return the concentration time profiles of reactants or products under specified environmental or experimental conditions [5–10]. However, the input parameters for KM may not be known *a priori*, and their determination can be challenging [11–14]. The deduction or constraint of model input parameters using model output is known as solving the inverse problem. In practice, researchers often utilize statistical approaches to solve the inverse problem with global optimization techniques [15–18]. Such techniques determine sets of parameter values, so-called fits, that lead to model outputs in agreement with previously acquired experimental data. In ill-posed problems, Berkemeier et al. 2021 [19] proposed the consideration of ensembles of sufficiently well-fitting parameter sets to extract information from the corresponding range of kinetic model solutions in underdetermined optimization problems. This approach is related to approximate Bayesian computation, a method for statistical inference that can be applied if the likelihood function is not known and the posterior distribution cannot be obtained directly [20]. This is often the case for computational or simulation-based models that are evaluated through calculation of a mechanism, like (bio-)chemical kinetic models [21, 22]. In approximate Bayesian computation, a probability density function is replaced by an artificial data set obtained through sampling of an approximate posterior distribution using a distance metric [23]. In this work, the approximate posterior distribution corresponds to the fit ensemble, i.e., kinetic parameter sets that lead to valid solutions matching experimental data within a specified error margin.

Quantitative structure–activity relationship (QSAR) models utilize the concept of molecular similarity to derive properties (e.g., chemical or biological) of new molecules from existing data, often through machine learning [24]. The models are generally trained on data derived from experimental measurements [25] or density functional theory (DFT) calculations [26–28]. Similarly to the acquisition of fit ensembles in global optimization, ensemble learning techniques allow the acquisition and utilization of multitudes of QSAR model predictions [29–31]. Such ensemble predictions have recently been utilized for uncertainty quantification, based on variance in predictions of Siamese neural networks [32].

Surrogate models (SM) are machine learning models that are trained on inputs and outputs of a template model. A SM can be used to substitute the template model in applications that benefit from low computational cost in exchange for slightly increased model uncertainty. Satisfactory model accuracy can be ensured by a sufficient size of the training data set, and therefore depends on the initial investment of computational resources [33]. SM have helped solving the issue of computational cost in many fields of research, such as in geoscientific and atmospheric modelling [34–40], chemical process engineering [41], water resources modelling [42, 43], or optimization in supply chain management [44]. SM can also aid inverse modelling approaches. Berkemeier et al. 2023 [33] showed that SM-supported fit ensemble acquisition greatly outperforms regular sampling with the kinetic multi-layer model of aerosol surface and bulk chemistry (KM-SUB) [5] in terms of acquired fits for a given computational effort. However, it remains unclear how SM uncertainty affects the reliability of inverse modelling techniques.

A kinetic model’s uncertainty can be based on model form uncertainty, i.e., concerning the underlying physics or chemistry, or model parametric uncertainty, i.e., concerning the knowledge of its input parameters [45]. Parametric uncertainty is often caused by the coupled nature of parameters or by underdetermination of the modelled system. Among model input parameters, we differentiate between kinetic parameters that define the physical and chemical properties of the modelled system (e.g., reaction rate coefficients), and parameters that define the environmental or experimental conditions (e.g., initial concentrations or temperature). When a model is evaluated for experimental conditions that differ from those for which its kinetic parameters were derived, model uncertainty may strongly increase [2]. This situation may arise in particular when the data underlying the model is limited, or when conditions in the laboratory experiment (e.g., a test reactor) deviate from the real-world application of interest (e.g., the atmosphere, an industrial plant, or an engine). Furthermore, when extrapolating a model to conditions outside its calibration range, not all fits in a fit ensemble may behave in the same way. This ensemble variance associated with a fit ensemble can be used to assess the model’s parametric uncertainty over a range of experimental conditions [19]. The ensemble variance at a specific set of experimental conditions may also be an indicator for parameter sensitivity, and of the potential to constrain the model if experimental data was available for these conditions. Thus, while data from any additional experiment may decrease the parametric uncertainty of a model, this process can be optimized by selecting experimental conditions associated with high ensemble variance. These conditions are most likely to constrain the underlying model and its physical and chemical parameters.

For experimenters, it is difficult to guess such optimal conditions *a priori*. As quantitative approaches to this problem, a number of methods and frameworks for targeted design of experiments (DOE) for uncertainty minimization have emerged over the past years, mostly in the fields of fuel combustion and computational fluid dynamics [46]. For this purpose, Bayesian experimental design methods have been proposed to maximize a utility function, e.g., through minimization of information entropy, a measure for the degree of disorder, diversity and dispersion [47]. DOE techniques have since then been continuously extended and improved, e.g., through the utilization of polynomial surrogate models [48], sensitivity entropy as a measure of the degree of dispersion of uncertainty sources of a model output [49], truncated Gaussian probability density functions [50] or surrogate model similarity methods [51]. For example, Lehn et al. successfully applied an iterative model-based experimental design framework based on the criterion of D-optimality [52] as well as polynomial chaos expansion [53] to identify optimal conditions for experimental measurements related to the auto-ignition of dimethyl ether [54]. Through integration of functions for dimension reduction, global sensitivity analysis, forward uncertainty quantification, model-analysis-based experimental design and model optimization, Zhou et al. developed a versatile computational framework (OptEx) to automatically find informative while independent experiments, and refine computational models [55]. Similar methods for so-called calibration experiment design optimization techniques have been developed and are applied in the fields of engineering and materials science [56, 57]. To our knowledge, however, such techniques had not yet been developed and applied to guide laboratory experiments in the fields of atmospheric and environmental multiphase chemistry.

Existing DOE methods are often based on optimality criteria to minimize the trace (A-Optimality), determinant (D-Optimality) or eigenvalue (E-Optimality) of the Fisher information matrix, and require knowledge of a likelihood function, given experimental data and uncertainty [52, 58]. To calculate the Fisher information matrix, derivatives of the likelihood function with respect to the model parameters must be obtained [59]. If automatic differentiation is not applicable to the model [60], the calculation of gradients through, e.g., finite differences [61], requires multiple model evaluations per maximum likelihood estimate and tested experimental condition [62]. In this work, we propose a new approach to the selection of optimal experiments. The numerical compass (NC) method treats experimental uncertainty implicitly through choice of acceptance conditions (e.g., thresholds) to derive a fit ensemble as representation of the underlying solution space. The approach represents a least-squares method for parameter estimation, in contrast to the more common maximum likelihood estimation methods [63]. The optimality criteria for the selection of experiments in our proposed method are formulated as statistical criteria, which we will refer to as *constraint potentials*. These are computationally inexpensive operations that only require one model evaluation per fit and tested experimental condition. The criteria can be specifically tailored to consider additional information associated with the fit ensemble, or specific properties of the model. In the proposed framework, we introduce two constraint potential metrics: one approximates the heterogeneity of models (i.e., posterior distribution samples) at different experimental conditions, and one that further explores the nature of constraint potentials with regards to individual kinetic parameters. The NC is used alongside the kinetic multi-layer model of aerosol surface and bulk chemistry (KM-SUB), and a neural network SM for it, to demonstrate its functionality in experiment design and inverse modelling. In addition to experiment design, we apply the NC to uncertainty quantification of machine learning quantitative structure–activity relationship (QSAR) models. The NC is used to explore molecular structures for which QSAR models exhibit a particularly high uncertainty and test whether this information can be used to suggest new training data that will increase model accuracy.

## Method

**Fig. 1:**
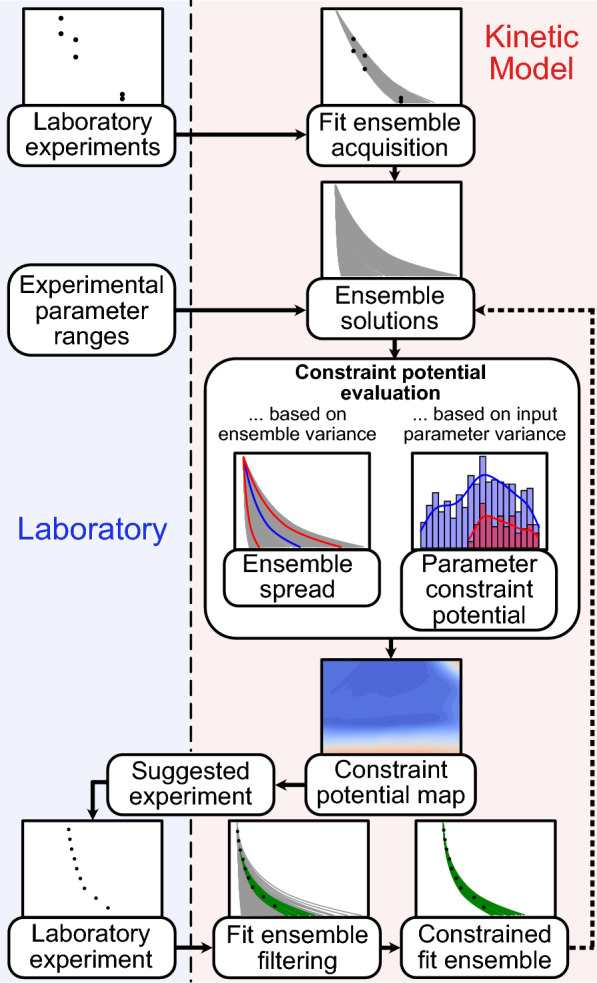
Workflow of the numerical compass (NC) method presented in this study. The method relies on exchange between laboratory experiments (left) and model calculations (right) to eliminate variance in model output. Data from laboratory experiments are used for the acquisition of a fit ensemble, which are kinetic parameter sets that lead to model outputs in agreement with the experimental measurements. Evaluating the model for the entire fit ensemble and over a defined range of experimental parameters yields sets of ensemble solutions that serve as the basis for all calculations with the NC. The NC offers two metrics for constraint potential evaluation: ensemble spread, and parameter (boundary) constraint potential (section Parameter boundary constraint potential). The metrics are used to build constraint potential maps, which highlight areas with large model output variance in the experimental parameter range. These experimental parameters are suggested as next experiment as they are likely to lead to rejection of a large number of fits during fit ensemble filtering. The NC can be used iteratively (dotted arrow), using the ensemble solutions of the constrained fit ensembles

We present the numerical compass (NC), a method for experiment prioritization and reduction of a model’s parametric uncertainty. The method requires a process model, data from previous laboratory experiments, and a set of variable experimental parameters that describe future experiments of interest. The individual steps of the proposed workflow are displayed in Fig. 1.

### Inverse modelling solutions and uncertainty

To estimate parametric uncertainty, inverse modelling can be extended to an ensemble of kinetic parameter sets that return sufficient agreement with experimental data [15, 19]. All possible sets of chemical and physical parameter values that lead to a sufficiently low residual between model output and experimental data, so-called fits, form the solution space of a kinetic model. In practice, we use a finite collection of fits, referred to as *fit ensemble*, as representation of the model solution space. Additional experimental data can help to narrow down the fit ensemble and thus decrease model parametric uncertainty.

### Operating principle

The NC is a framework to optimize the deduction or constraint of kinetic parameters with experiments. In general, the information gained from new experimental data can be used to reject fits from a fit ensemble. The NC finds experimental conditions with the highest constraint potential, optimizing the reduction of model solution space and hence model parametric uncertainty. For this purpose, the method computes ensemble solutions under experimental conditions that have not been considered previously, and determines the ensemble variance under these conditions. We present two metrics evaluating the ensemble variance, the *ensemble spread* of model solutions (section Ensemble spread) and the *parameter (boundary) constraint potential* (section Parameter boundary constraint potential). By sampling the space of feasible experiments, *constraint potential maps* (section Constraint potential maps) of these metrics are obtained. Maxima on these maps represent prospective experiments that are most likely to achieve large constraints of the model. After fit ensemble filtering based on the new experimental data, the NC method can be repeated to suggest the next experiment. In this study, we simulate the suggested laboratory experiments with the model KM-SUB to showcase the alternating application of the NC with laboratory experiments. For more detailed and mathematical definitions of process models, their solution space, as well as fit ensembles and ensemble solutions, see Additional file 1: Note S1.

### Ensemble spread

The ensemble spread is a measure for the variance between a multitude of model predictions. Resembling similar concepts in weather and climate forecasting [64], we calculate the ensemble spread (ES) as:

1 $$\begin{aligned} \text {ES} = \frac{\int ({\overline{Z}}(x)+\sigma _Z(x)) dx - \int ({\overline{Z}}(x)-\sigma _Z(x)) dx}{\int {\overline{Z}}(x) dx} \end{aligned}$$

where $(x_m)_{m=1,...,n_z}$ is the sequence of independent variables associated with the output sequence $(z_m)_{m=1,...,n_z}$, and $\int {\overline{Z}}$, $\int {\overline{Z}}+\sigma$ and $\int {\overline{Z}}-\sigma$ are integrals of the interpolated sequences $(\overline{Z_m})_{m=1,...,n_z}$, $(\overline{Z_m} + \sigma _m)_{m=1,...,n_z}$ and $(\overline{Z_m} - \sigma _m)_{m=1,...,n_z}$ for $n_z$ model outputs with an ensemble mean $\overline{Z_m}$ and ensemble standard deviation $\sigma _m$ (Additional file 1: Note S2).

**Fig. 2:**
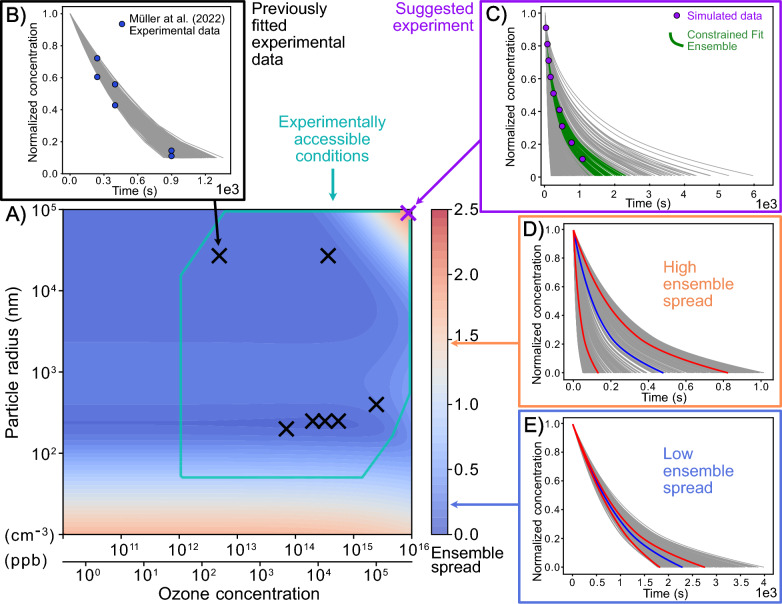
Constraint potential map obtained with the numerical compass (NC) method. The contour map in **A** shows an exemplary constraint potential map using the ensemble spread metric. Model calculations are obtained with KM-SUB on a 100$\times$100 grid of two experimental parameters, ozone concentration and particle radius, and for a fit ensemble of 500 fits. The teal box frames the area of experimentally accessible conditions with regards to particle radius, ozone concentration and predicted experiment duration (Additional file 1: Note S4). Black crosses in **A** mark the experimental conditions of available experimental data that were used to obtain the fit ensemble (cf. Fig. 3) and **B** shows the ensemble solution (gray lines) in comparison to one of these experimental data sets (blue markers). The purple cross in **A** represents the ensemble spread maximum within experimental accessibility and thus the recommended experiment. **C** Illustrates the ensemble solution at this ensemble spread maximum. New experimental data from the recommended experiment (purple markers) are used to obtain the constrained fit ensemble (green lines) through rejection of fits. **D**, **E** Showcase ensemble solutions with a high ensemble spread of 1.446 and a low ensemble spread of 0.234, respectively. Here, colored lines visualize the mean of the ensemble solution (blue line) and the mean ± 1 standard deviation (red lines)

In short, the ensemble spread describes the area enclosed by the curves of the ensemble mean ± its standard deviation, normalized by the area under the ensemble mean curve. Visualizations of the ensemble spread as constraint potential metric are provided in Fig. 2D, E. A large ensemble spread is generally associated with a larger fraction of rejected fits during fit ensemble filtering.

### Parameter boundary constraint potential

The parameter (boundary) constraint potential allows an extension of the method to constraint potentials of individual kinetic parameters. The metric quantifies the potential narrowing of an individual parameter’s boundaries in the constrained fit ensemble.

In brief, the parameter constraint potential is calculated by iterating over predictions in an ensemble solution. In each iteration, one prediction is considered as the hypothetical result of an experiment. Based on this prediction, we calculate a hypothetical constrained fit ensemble and derive the distribution of the kinetic parameter in the remaining fits. The kinetic parameter’s boundaries in this distribution are normalized by its boundaries in the original fit ensemble to compute a numerical value for the parameter’s constraint potential.

More specifically, we determine the subset C of the fit ensemble FE. C contains all fits that lead to model solutions within acceptance threshold $\theta$ in comparison to the model solution of fit FE_l_ that is selected as hypothetical measurement in the iteration *l* over all predictions in the ensemble solution:

2 $$\begin{aligned} \text {C}_{l} = \{\text {FE}_r: \Delta (\text {ENS}_l, \text {ENS}_r) < \theta \} \end{aligned}$$

where ENS_l_ and ENS_r_ are the model solutions using fits FE_l_ and FE_r_ in the evaluated ensemble solution (ENS). Hence, we obtain one subset C_l_ in each iteration. If every solution in the ensemble is evaluated as hypothetical experimental result in turn, $n_{\text {FE}}$ subsets are generated for every ensemble solution, where *n*_FE_ is the number of elements in the fit ensemble . The parameter constraint potential (PCP) for a specific parameter $\lambda _p$ and ensemble solution is then defined as:

3 $$\begin{aligned} \text {PCP}_p = \sum _{l=1}^{n_\text {FE}}{(\text {Q}{} \textit{5}_{\lambda _p,l} - \min (\lambda _p)) + (\max (\lambda _p) - \text {Q}{} \textit{95}_{\lambda _p,l})} \end{aligned}$$

where Q$\textit{5}_{\lambda _p,l}$ and $\text{Q}\textit{95}_{\lambda _p,l}$ are the 5- and 95-percentiles of the distribution of $\lambda _p$ in subset C_l_, respectively. $\min (\lambda _p)$ and $\max (\lambda _p)$ are the global minimum and maximum of the selected kinetic parameter in the entire fit ensemble.

Note that the computational effort associated with this method is large due to the pairwise comparison of all predictions in an ensemble solution. Therefore, we suggest an approximation based on a reduced sample density. A detailed definition of the parameter constraint potential with reduced sample density is presented in Additional file 1: Note S3 and visualized in Additional file 1: Fig. S1.

Note that we can apply the same principle of forming subsets of the fit ensemble based on their behavior under test conditions, to constrain model uncertainty at a specific target condition (Additional file 1: Fig. S2). This can be of high practical relevance for situations where laboratory experiments must be performed outside the typical conditions of the target application, a common problem in the fields of atmospheric chemistry and chemical technology.

### Constraint potential maps

The application of a metric for model constraint potential on a range of ensemble solutions (one for each tested experimental condition) can be visualized in a constraint potential map. This map is a *n*-dimensional hypersurface, where *n* is the number of varied experimental parameters, and whose maxima represent experimental conditions favorable for constraint of the underlying model. An example for a constraint potential map is presented for two varied experimental parameters and the ensemble spread metric in Fig. 2. For further information on the chemical system (oleic acid ozonolysis) and the variable experimental parameters (particle radius, ozone concentration), as well as a description of the restrictions regarding experimental accessibility applied in this work, see section Kinetic multi-layer model and neural network surrogate model, Additional file 1: Note S4, and Additional file 1: Fig. S3. Note that while we evaluate a full grid of combinations of experimental parameters for the purpose of testing and visualization, the constraint potential metrics can similarly be used as an objective function of an optimization algorithm to reduce the required computational effort.

### Kinetic multi-layer model and neural network surrogate model

In this study, we use the kinetic multi-layer model of aerosol surface and bulk chemistry (KM-SUB) [5] along with experimental data of the heterogeneous ozonolysis of oleic acid from the literature. However, the NC method can be used with any process model and underlying chemical or physical system. Detailed information about KM-SUB can be found in previous publications [5, 12]. In brief, KM-SUB is a chemical flux model that explicitly describes gas diffusion, accommodation of gas molecules to surfaces, surface-bulk exchange, bulk diffusion, as well as chemical reaction at the surface and in the bulk of a condensed phase. The resulting set of ordinary differential equations is solved numerically. KM-SUB input parameters include initial concentrations, chemical reaction rate coefficients, and mass transport coefficients, and are presented in Table 1. KM-SUB outputs are the concentration profiles over space and time for all chemical species.

For the training of neural network surrogate models, KM-SUB output is simplified to nine points of reaction progress, i.e., the time required to reach 90 %, 80 %, 70 %, 60 %, 50 %, 40 %, 30 %, 20 % and 10 % of the total number of oleic acid (OL) in a single aerosol particle, $N_{\text {OL},0}$. For comparability, we represent the output of the full KM-SUB model in this study in the same way. We train a fully-connected, feed-forward neural network on $1\times 10^6$ KM-SUB outputs as training data. For further information on training of the surrogate model see Berkemeier et al. 2023 [33] and Additional file 1: Note S5.

The NC method requires evaluation of the underlying process model during fit ensemble acquisition and during calculation of ensemble solutions (Fig. 1). In this study, we test and compare three different approaches: using KM-SUB for both steps (KM-only), using an SM of KM-SUB for both steps (SM-only), and a KM/SM-hybrid approach, in which KM-SUB is used for fit ensemble acquisition and the SM to obtain ensemble solutions. Fit ensemble acquisition is achieved by random sampling of kinetic input parameters with the KM or SM within the parameter boundaries in Table 1, using a mean square logarithmic error (MSLE) and an acceptance threshold $\theta$ = 0.0105 to determine sufficient agreement with experimental data. For the specifications of fit ensemble acquisition and error calculation in this study, see Additional file 1: Note S6.

**Table 1:** KM-SUB kinetic and experimental input parameters

Parameter	Lower boundary	Upper boundary	Description
$k_{\text {SLR}}$	1.0 $\times$ 10^-15^	1.0 $\times$ 10^-8^	Rate coefficient of OL+O_3_ surface reaction (cm^3^ s^-1^)
$k_{\text {BR}}$	1.0 $\times$ 10^-20^	1.0 $\times$ 10^-11^	Rate coefficient of OL+O_3_ bulk reaction (cm^3^ s^-1^)
$D_{\text {b,O3}}$	1.0 $\times$ 10^-11^	1.0 $\times$ 10^-5^	Bulk diffusion coefficient of ozone (cm^2^ s^-1^)
$D_{\text {b,OL}}$	1.0 $\times$ 10^-12^	1.0 $\times$ 10^-6^	Bulk diffusion coefficient of oleic acid (cm^2^ s^-1^)
$H_{\text {cp,O3}}$	5.0 $\times$ 10^-6^	5.0 $\times$ 10^-3^	Henry’s law solubility coefficient of ozone (mol cm^-3^ atm^-1^)
$\tau _{\text {d,O3}}$	1.0 $\times$ 10^-9^	1.0 $\times$ 10^-2^	Desorption lifetime of O_3_ (s)
$\alpha _{\text {s,0,O3}}$	1.0 $\times$ 10^-4^	1	Surface accommodation coefficient of ozone on an adsorbate-free surface ( )
$r_{\text {p}}$	2.5 $\times$ 10^-6^	1.0 $\times$ 10^-3^	Particle radius (cm)
$[\text{O}_3]_{\text {g,0}}$	1.0 $\times$ 10^11^	1.0 $\times$ 10^15^	Initial gas phase number concentration of ozone (cm^-3^)
$[\text{OL}]_{\text {b,0}}$	1.0 $\times$ 10^19^	2.0 $\times$ 10^21^	Initial bulk number concentration of oleic acid (cm^-3^)

The respective lower and upper boundaries indicate the initial constraints of the fit ensemble and an estimate of experimentally accessible conditions in a laboratory for atmospheric aerosol chemistry

### Quantitative activity structure relationship models and ensemble learning

In addition to experiment design, the NC can be utilized for uncertainty quantification of QSAR models. We use a re-trained version of the CNN_Tabor_nosulf model from Krüger et al. [28], a convolutional neural network model predicting reduction potentials based on SMILES molecular representations of 69,599 quinones from the Tabor et al. [65] data set, excluding quinone structures that contain sulfate functional groups. The models are trained on identical hyper-parameters as in the original study, but using 10-fold instead of 5-fold cross-validation. In this application, the ensemble solution utilized by the NC refers to multiple cross-validation models that are trained on different subsets of the training data. We calculate a non-normalized ensemble spread of predicted reduction potentials for a set of autogenerated quinone structures.

## Results and discussion

### Acquisition of fit ensembles

**Fig. 3:**
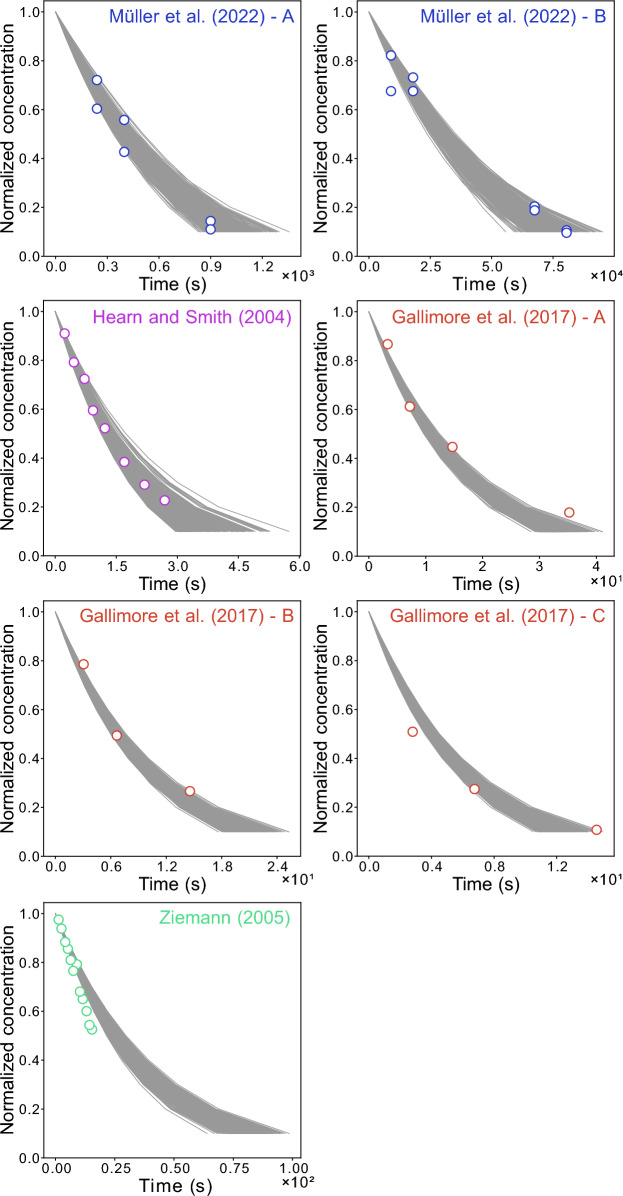
Ensembles of kinetic multi-layer model of aerosol surface and bulk chemistry (KM-SUB) outputs (*n*_FE_ = 500, gray lines) with a mean square logarithmic error (MSLE) < 0.0105 in comparison with seven literature data sets (markers) of oleic acid aerosol ozonolysis displayed as normalized oleic acid concentrations ($N_{\text {OL},t}/N_{\text {OL},0}$)

We demonstrate the applicability of the numerical compass (NC) method for the heterogeneous ozonolysis of oleic acid aerosols using the kinetic multi-layer model of aerosol surface and bulk chemistry (KM-SUB), and a neural network surrogate model (SM) for it. Both models map seven kinetic and three experimental input parameters (Table 1) onto the concentration-time profile of oleic acid. For each model, we obtained fit ensembles (*n*_FE_=500) in compliance with seven experimental data sets [8, 66–68] as shown in Fig. 3. Each kinetic parameter set in the fit ensemble is associated with one model output (gray lines) for each experimental condition. Both fit ensembles (of KM-SUB and the SM) have a minimal mean-squared logarithmic error (MSLE) of 0.0085; the median MSLE are 0.0102 for KM-SUB and 0.0099 for the SM.

### Ensemble spread

**Fig. 4:**
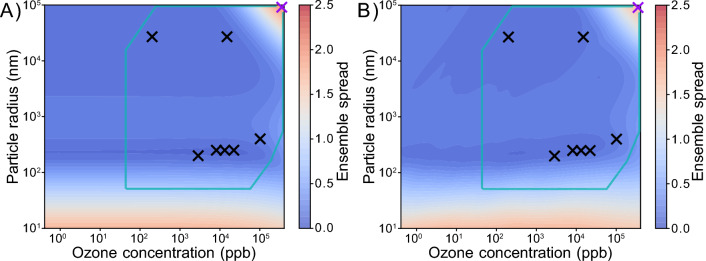
Constraint potential maps for the ensemble spread, evaluated by **A** KM-SUB (KM-only approach) and **B** SM, based on the KM-SUB fit ensemble (KM/SM-hybrid approach). The teal box outlines conditions for feasible experiments. Black crosses represent the experimental parameters of the seven real experiments that are used for the initial acquisition of the fit ensemble. Purple crosses represent the ensemble spread maximum in each grid with satisfied experimental constraint conditions

The ensemble spread aims for general minimization of the solution space of a model. Figure 4 displays constraint potential maps for the ensemble spread metric and the variable experimental parameters of particle radius $(r_{\text {p}}$) and ozone concentration ($[\text {O}_3]_{\text {g,0}}$). The conditions associated with the experimental data used to obtain the fit ensemble (black crosses) are, naturally, located in areas of low ensemble spread. Maxima of the ensemble spread, i.e., regions associated with large model variance, occur at very low particle radii (< 50 nm), and for the combination of large radii (> 10 $\mu$m) with high ozone concentrations (> 100 ppm). The constraint potential maps obtained with the KM-only approach (panel A) and the KM/SM-hybrid approach (panel B) appear similar overall. The absolute ensemble spread maxima are both located at maximal particle radii and ozone concentrations (purple crosses). As main difference, isopleths appear less smooth for the SM. A constraint potential map of the SM-only approach is displayed in Additional file 1: Fig. S7. The computationally less expensive SM-only method leads to slightly larger differences to the KM-SUB constraint potential map. In particular, the ensemble spread maximum at low particle radii is less pronounced.

### Parameter boundary constraint potential

**Fig. 5:**
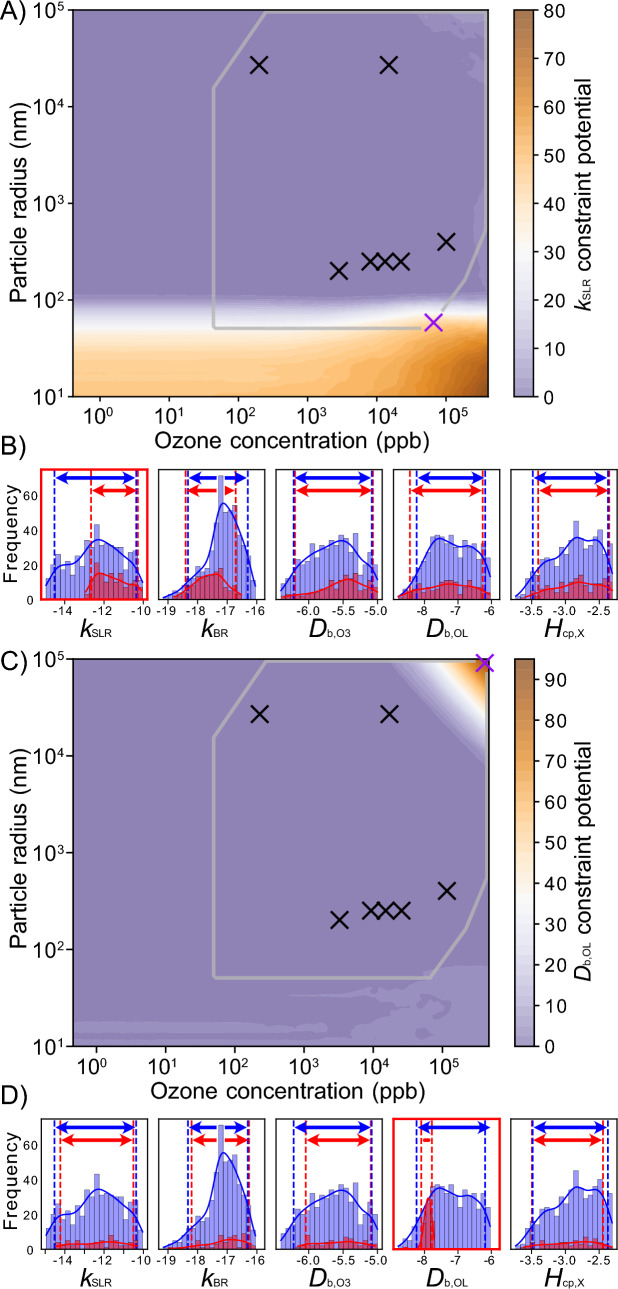
Constraint potential maps for the kinetic parameters **A** $k_{\text {SLR}}$ and **C** $D_{\text {b,OL}}$ obtained with KM-SUB. The gray box outlines conditions for feasible experiments. Black crosses represent the experimental parameter sets of the seven real experiments that are used for the initial acquisition of the fit ensemble. The purple crosses represent the parameter constraint potential maxima with satisfied experimental constraint conditions. The suggested experimental conditions are used to obtain synthetic experimental data by evaluating KM-SUB for the best fit in the KM-SUB fit ensemble. Frequency distributions of five kinetic parameters are shown and highlighted for **B** $k_{\text {SLR}}$ and **D** $D_{\text {b,OL}}$ in the KM-SUB fit ensemble before (blue) and after (red) fit filtering with acceptance threshold $\theta =$ 0.0105. Blue and red dotted lines and arrows visualize the 5-95 percentile range of each distribution

In addition to the ensemble spread, we apply the NC using both models with the parameter constraint potential (section Parameter boundary constraint potential). This method aims for a minimization of a chosen kinetic parameter’s uncertainty range in the solution space, approximated through its 5-95 percentile range in the fit ensemble. Figure 5A and C display parameter constraint potential maps for the kinetic parameters $k_{\text {SLR}}$ and $D_{\text {b,OL}}$, respectively. The maximum of the $k_{\text {SLR}}$ constraint potential matches the maximum of the ensemble spread at low particle radii in Fig. 4, whereas the maximum of the $D_{\text {b,OL}}$ constraint potential matches the maximum of the ensemble spread at large radii and high ozone concentrations. Hence, high ensemble spreads appear to be necessary but not sufficient conditions for high parameter constraint potentials.

We simulate the suggested experiments with KM-SUB, using the best fit in the KM-SUB fit ensemble as simulated truth. Under consideration of the original data and the new synthetic experiment, we filter the fit ensembles using the MSLE threshold of $\theta =$ 0.0105. Figure 5B and D show frequency distributions of five kinetic parameters in the fit ensemble before (blue) and after (red) fit filtering. The experiments suggested by the constraint potential metrics achieve a significant reduction in the 5-95 percentile range for their associated parameters, $k_{\text {SLR}}$ and $D_{\text {b,OL}}$, respectively. Simultaneously, constraints are achieved for other parameters, e.g., $k_{\text {BR}}$ (Fig. 5B), following the similarity between the parameter constraint potential maps (Additional file 1: Fig. S8A, D, G, J). Parameter constraint potential maps and simulated constraints for the SM-only approach (Additional file 1: Fig. S9) are very similar to those using the KM-only approach.

### Empirical testing

The NC can be applied repeatedly to narrow down model solutions in iterative fashion. Here, we simulate this procedure using synthetic experimental data, which is obtained by assuming that a single fit from the fit ensemble is the true solution of the modelled system (the *simulated truth*). The simulation is repeated for each fit in the ensemble as simulated truth. Detailed information on the simulation of experimental data is presented in Additional file 1: Note S7.

**Fig. 6:**
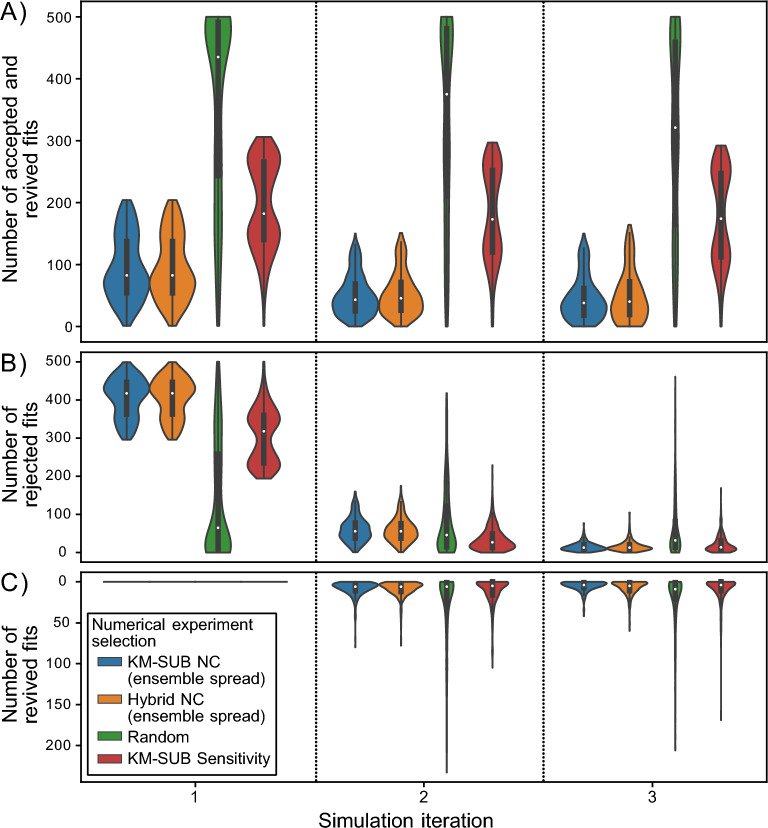
Number of fits that are **A** accepted, **B** rejected and **C** revived based on synthetic experimental data in three iterations of the numerical compass (NC) method. Numbers are based on statistics for *n* = 500 simulations, where each fit in the KM-SUB fit ensemble is once selected as simulated truth. Medians are shown as white markers, interquartile ranges as vertical wide black lines and 1.5 $\times$ interquartile ranges as narrow black lines. While experiment simulation (via KM-SUB) and fit filtering (of the KM-SUB fit ensemble, absolute MSLE threshold, $\theta =$ 0.0105) are identical for all approaches, we compare different numerical selection methods of experiments: KM-only NC (blue), KM/SM-hybrid NC (orange), random selection of experiments (green) and parameter sensitivities of the KM (red). The simulation is performed on a reduced 10$\times$10 grid of experimental conditions within the usual ranges. Fit ensemble constraints are significantly larger when experiments are selected using the NC. While the two models utilized for its evaluation lead to very similar fit ensemble constraints, the random and sensitivity-based selection of experiments perform significantly worse

Figure 6 shows the statistics of a total of 500 of these simulations with three iterations of the NC, and compares the performance of four numerical experiment selection methods: ensemble spread using KM-SUB (blue), ensemble spread using the KM/SM-hybrid approach (orange), random selection (green), and total sensitivities with respect to KM-SUB parameters (red, Additional file 1: Note S8). Figure 6A shows the decreasing number of accepted fits in the fit ensemble. The median numbers of remaining fits after each of the three iterations are (82.5, 43, 38) for the KM-SUB ensemble spread, (82.5, 45.5, 40) for the KM/SM-hybrid ensemble spread, (435, 373, 320.5) for the random selection, and (182, 172.5, 173.5) for the sensitivity-based experiment selection.

Hence, empirically, the NC leads to a significantly larger constraint of the fit ensemble compared to parameter sensitivity maximization or random selection, irrespective of using the full KM or the SM-assisted hybrid approach. Additional file 1: Figures S11–S14 show examples of individual trajectories of the NC, i.e., simulations including numerical experiment selection, synthetic experimental data generation, and fit filtering. We find that in contrast to constraint potentials maps, sensitivity maps barely change throughout the iterations of a simulation, and suggested experiments are usually the grid points closest to a persistent sensitivity maximum (Additional file 1: Fig. S15). Consequently, only the first sensitivity-guided experiment leads to a significant constraint of the fit ensemble and, while the performance of the sensitivity-guided method is better than random selection, it performs worse than the ES-guided method of the NC.

Spinning the idea of Fig. 6 further, we can ask: what are the ideal experimental conditions in such a simulation of synthetic experiments? We thus perform a “brute-force” simulation: we repeat the workflow of simulating laboratory experiments for each simulated truth (cf. Fig. 6), but do so for every experimental condition. Instead of the full distribution, we report the median number of rejected fits and plot the results in similar fashion to the constraint potentials into a 2D map (Additional file 1: Fig. S16B). We find that this map is strongly congruent with the ES map, showing empirically that the experimental conditions associated with the ES maximum are optimal to constrain a fit ensemble. We conducted the same analysis using the PCP metric with similar outcomes, finding major similarities between PCP maps and the maps of reduction of 5-95-percentile ranges for individual parameters in the brute-force simulation, but not to all partial sensitivity maps of individual kinetic parameters (Additional file 1: Fig. S8). Of course, this analysis assumes that there are fits in the fit ensemble that resemble the true solution, which must be ensured when using the compass method by sufficient sampling of the solution space.

Accurate representation of the solution space, especially in the light of experimental error, is contingent on the choice of the acceptance threshold $\theta$. If $\theta$ is set too low, a correct solution may be discarded due to incompatibility with a faulty experimental data set. We select a $\theta$ in this study so that visual agreement between the scatter in experimental data with the spread of the fit ensemble is achieved. The selection of an appropriate filter threshold is important when quantitative statistical conclusions ought to be drawn for general uncertainty quantification. However, in this context of model optimization or uncertainty minimization through experiments, information is derived by relative comparison of different experimental conditions. This makes the choice of acceptance thresholds for the initial fit acquisition one of practical nature, for example with regards to computational cost [69]. In approximate Bayesian computation, crucial steps like the selection of an acceptance threshold can not be based on general rules, but require testing and evaluation of the performances in the investigated system [70]. Repeating the calculations based on a fit ensemble with an acceptance threshold of $\theta$ = 0.021, we found no significant changes in the appearance of constraint potential maps and in the conditions of suggested experiments (Additional file 1: Fig. S17). While absolute values of constraint potential metrics naturally increase with a wider scatter of the ensemble solutions, we find that relative differences between experimental conditions and the locations of constraint potential maxima, denoting suggested experiments, persist across a wide range of acceptance thresholds.

### Application to quantitative structure activity relationship (QSAR) model training

**Fig. 7:**
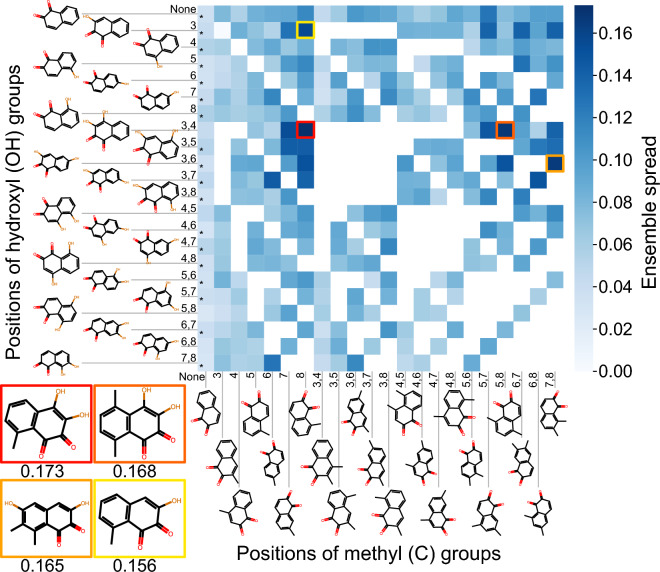
Heatmap of the non-normalized ensemble spread of QSAR model ensemble predictions for reduction potentials of generated quinone structures based on the template quinone 1,2-naphthoquinone with a maximum of two hydroxyl and methyl groups at varying positions. Ensemble predictions are obtained through 10-fold cross-validation models trained on a data set of 69,599 quinones. Fields marked with ‘*’ are quinones that are present in the training data set. White fields are impossible quinone structures. The molecular structures associated with the four largest ensemble spread values are shown in the bottom left

Figure 7 shows an exemplary result for quinone structures based on the template 1,2-naphthoquinone which is relevant for atmospheric chemistry and health due to its large reduction potential and ability to undergo redox-cycling. A variety of structures with one or multiple hydroxyl groups is present in the QSAR model’s training data, visualized through asterisks in the fields of the heat map. These structures are naturally associated with low ensemble spread values, an indicator for accurate predictions of the QSAR model. Among the newly-generated structures, significant differences in the ensemble spread are observed. In the presented example, structures with a methyl group at position 8, or hydroxyl groups at positions 3 and 4, lead to overall large ensemble spread values of the ensemble predictions. Structures associated with a large ensemble spread may have a larger potential to improve the accuracy of the QSAR models when added to the training data. In basic testing, we find that adding batches of molecules with a high ensemble spread to the model training data generally leads to a much larger improvement of the model compared to adding molecules with a low ensemble spread (Additional file 1: Fig. S18). However, randomly-chosen batches of molecules perform nearly as well, which indicates that more research is needed to optimize the usage of the NC in QSAR applications.

## Conclusion

This study demonstrates the application of computational models to guide experiment design and prioritization based on the anticipated reduction of a model’s solution space. The method extrapolates current ensemble solutions to conditions of potential future experiments and identifies conditions under which ensemble variance, and thus model parametric uncertainty is largest. In comparison with random selection and selection of experiments associated with maximum sensitivities of kinetic parameters, the reduction of fits in the fit ensemble is much larger for the numerical compass (NC) guided selection of experiments. A disadvantage we find for parameter sensitivities is their lack of variation across the fit ensemble, which makes the sensitivity-guided method mostly agnostic of prior information from experiments.

In contrast to common DOE methods, our proposed statistical approach to experiment design does not require the calculation of Fisher information matrices. This can be advantageous when the model does not permit automatic differentiation or when the computation of numerical gradients is prohibited by computational cost. Furthermore, the novel method is transparent and intuitive: constraints are defined as simple statistical criteria and applied to a tangible fit ensemble, which approximates the solution space. After optimization, the fit ensemble can be used as estimate for the remaining uncertainty of the model solution [19]. The approach can be easily integrated into existing modelling workflows using least-squares parameter estimation and thus offers a low-level entry to experiment design for researchers from various fields.

We find that our method returns near-identical results irrespective of choice of model (KM and SM), fit ensemble (KM and SM fit ensemble) and acceptance threshold for fit ensemble acquisition. This shows the robustness of the method and gives evidence that the properties of the solution space are well-represented by fit ensembles in this study.

Furthermore, the method allows for incorporation of additional information or can be tailored to objectives respective to a specific system, such as chemical kinetic regimes, constraints of specific parameters, or constraints on a specified target condition. We demonstrate this approach by evaluating constraint potentials for individual kinetic parameters (parameter constraint potential; Fig. 5) and by determining optimal experiments for the minimization of model uncertainty under the specific conditions relevant for atmospheric chemistry (target constraint potential; Additional file 1: Fig. S2).

The versatility of the NC is demonstrated through its application on uncertainty quantification of a QSAR model for the prediction of quinone reduction potentials. In analogy to the conditions of kinetic experiments, molecular structures that are associated with high model uncertainty represent potential candidates for future model training. This optimization of training data through uncertainty quantification may be especially useful in organic chemistry, where large quantities of molecules can be generated for computationally-costly density functional theory calculations. In basic tests, we find a correlation of the uncertainty of molecules that are added to the training data and the resulting QSAR model accuracy. However, compared with random selection, only a slight improvement in model accuracy is achieved. Thus, application of the NC for the optimization of QSAR models requires further research and will be the subject of future studies.

The computational effort of the NC can be strongly reduced by training a neural network surrogate model (SM), with nearly identical results. After consideration of the computational effort of SM training, and for the system at hand, we observe an acceleration of the evaluation of the NC by a factor of $\sim$5 using a KM/SM-hybrid approach, and an acceleration by a factor of $\sim$7.5 using only the SM (Additional file 1: Note S9). While SM for multiphase kinetic models have already proven useful in forward modelling applications [33], we here further demonstrate their utility in an inverse modelling approach.

For the kinetic multi-layer model of aerosol surface and bulk chemistry (KM-SUB) and the heterogeneous ozonolysis of oleic acid, the NC suggests experiments with either very small particles (< 50 nm) or with exceptionally large particles ($\approx 100$ $\mu$m) and high ozone concentrations ($\approx 1000$ ppm) (section Ensemble spread). The first suggestion seems logical: experiments with nano-sized particles of oleic acid have not been conducted and extrapolation to these conditions will be associated with model uncertainty. The method predicts that measurements using nano-sized particles would help especially to constrain the surface reaction rate coefficient $k_\text {SLR}$. The second suggestion of the NC may seem counter-intuitive, as these large particle—high ozone conditions are far away from atmospheric relevance. In fact, these experiments likely offer a constraint on the diffusion coefficient of oleic acid, $D_\text {b,OL}$, a parameter that is rather unimportant under typical atmospheric conditions. Note, however, that the simple model used in this analysis does not consider changes in $D_\text {b,OL}$ upon formation of oxidation products.

Overall, this analysis of the oleic acid—ozone reaction system shows that additional experiments measuring the loss of oleic acid under conditions typical for the atmosphere will not improve our knowledge of this well-studied system any further. More extreme conditions are needed to narrow down the model solution space, however, this will not come with an improvement of the predictive power of our models for atmospheric conditions (other than small nano-particles). Conversely, any solution in the fit ensemble obtained in this study and in Berkemeier et al. 2021 [19] should perform well under atmospherically-relevant conditions. More knowledge about the system can also be derived by changing the experimental observable. For the heterogeneous ozonolysis of alkenes, for example, product analyses have recently provided additional constraints for kinetic models [68, 71]. Extending the NC from experimental conditions to experimental observables will be a subject of future studies.

### Supplementary Information

**Additional file 1: Note S1.** Equations for process models, fit ensembles and prediction ensembles. **Note S2.** Equations for ensemble mean and standard deviation. **Note S3.** Parameter boundary constraint potential metric with reduced sample density. **Note S4.** Oleic acid ozonolysis system applied in this study. **Note S5.** Surrogate model training. **Note S6.** Fit ensemble acquisition with KM-SUB and SM. **Note S7.** Uncertainty calibration and simulated experiments. **Note S8.** Sensitivity analysis. **Note S9.** Computational effort. **Figure S1.** Visualization of the parameter constraint potential metric. **Figure S2.** Constraint potential map for the target constraint potential evaluated by KM-SUB. **Figure S3.** Restrictions for constraint potential maps with regards to experimental feasibility. **Figure S4.** Contrariwise cross evaluation of the KM-SUB and SM fit ensembles. **Figure S5.** Scatter plot matrix of the KM-SUB fit ensemble. **Figure S6.** Scatter plot matrix of the SM fit ensemble. **Figure S7.** Constraint potential maps for the ensemble spread, evaluated by KM SUB and SM. **Figure S8.** Comparison of methods to approximate constraints for individual parameters. **Figure S9.** Parameter constraint potential maps evaluated by KM-SUB and the SM. **Figure S10.** Visualization of the uncertainty calibration method. **Figure S11.** Simulated trajectories for iterative NC application. **Figure S12.** Simulated trajectories for iterative NC application. **Figure S13.** Simulated trajectories for iterative NC application. **Figure S14.** Simulated trajectories for iterative NC application. **Figure S15.** Maps of total KM-SUB sensitivity for three iterations of an example simulation for the NC. **Figure S16.** Ensemble spread, median brute-force simulated constraints and total KM-SUB parameter sensitivities. **Figure S17.** Constraint potential map for the ensemble spread evaluated by the SM with a fit ensemble acceptance threshold of 0.021. **Figure S18.** Effect of ensemble spread in additional training data on the QSAR model accuracy of a newly trained model.

## Data Availability

The data is openly available at 10.17617/3.D5PCQK.

## Abbreviations

PCP: Parameter boundary constraint potential
DOE: Design of experiments
ENS: Ensemble solution
ES: Ensemble spread
FE: Fit ensemble
KM: Kinetic model
KM-SUB: Kinetic multi-layer model of aerosol surface and bulk chemistry
MSLE: Mean-squared logarithmic error
NC: Numerical compass
OL: Oleic acid
QSAR: Quantitative Structure–Activity Relationship
SM: Surrogate model

## Code availability

The source code is openly available at 10.17617/3.D5PCQK. The NC is available as package for the programming language Julia (*KineticCompass*) at https://gitlab.mpcdf.mpg.de/mkruege/kineticcompass.
