# Supplementary material for: A numerical compass for experiment design in chemical kinetics and molecular property estimation
Source: J Cheminform. 2024 Mar 22;16:34. doi: 10.1186/s13321-024-00825-0 (PMC10960421; doi:10.1186/s13321-024-00825-0)
Supplement: Supplementary file 1 — Additional file 1: Note S1. Equations for process models, fit ensembles and prediction ensembles. Note S2. Equations for ensemble mean and standard deviation. Note S3. Parameter boundary constraint potential metric with reduced sample density. Note S4. Oleic acid ozonolysis system applied in this study. Note S5. Surrogate model training. Note S6. Fit ensemble acquisition with KM-SUB and SM. Note S7. Uncertainty calibration and simulated experiments. Note S8. Sensitivity analysis. Note S9. Computational effort. Figure S1. Visualization of the parameter constraint potential metric. Figure S2. Constraint potential map for the target constraint potential evaluated by KM-SUB. Figure S3. Restrictions for constraint potential maps with regards to experimental feasibility. Figure S4. Contrariwise cross evaluation of the KM-SUB and SM fit ensembles. Figure S5. Scatter plot matrix of the KM-SUB fit ensemble. Figure S6. Scatter plot matrix of the SM fit ensemble. Figure S7. Constraint potential maps for the ensemble spread, evaluated by KM SUB and SM. Figure S8. Comparison of methods to approximate constraints for individual parameters. Figure S9. Parameter constraint potential maps evaluated by KM-SUB and the SM. Figure S10. Visualization of the uncertainty calibration method. Figure S11. Simulated trajectories for iterative NC application. Figure S12. Simulated trajectories for iterative NC application. Figure S13. Simulated trajectories for iterative NC application. Figure S14. Simulated trajectories for iterative NC application. Figure S15. Maps of total KM-SUB sensitivity for three iterations of an example simulation for the NC. Figure S16. Ensemble spread, median brute-force simulated constraints and total KM-SUB parameter sensitivities. Figure S17. Constraint potential map for the ensemble spread evaluated by the SM with a fit ensemble acceptance threshold of 0.021. Figure S18. Effect of ensemble spread in additional training data on the QSAR model accuracy of a newly t [file 13321_2024_825_MOESM1_ESM.pdf]

Supplementary Information for "A numerical compass for experiment design in chemical kinetics and molecular property estimation"

Matteo Krüger[1], Ashmi Mishra[1], Peter Spichtinger[2], Ulrich Pöschl[1], and Thomas Berkemeier[1]

[1]Multiphase Chemistry Department, Max Planck Institute for Chemistry, Hahn-Meitner-Weg 1, 55128 Mainz, Germany
[2]Institute for Atmospheric Physics (IPA), Johannes Gutenberg University, Johann-Joachim-Becher-Weg 21, 55128 Mainz, Germany

Correspondence: Thomas Berkemeier (t.berkemeier@mpic.de)

Supplementary Note 1: Equations for process models, fit ensembles and prediction ensembles

The numerical compass (NC) method can be evaluated with any predictive process model M:

$$\mathrm{M}: \mathbb{R}^{n_\lambda + n_\mathrm{s}} \to \mathbb{R}^{n_z} \tag{S.1}$$

$$\mathrm{M}(\lambda, \mathrm{s}) = z \tag{S.2}$$

where λ are the kinetic input parameters $(\lambda_p)_{p=1,\dots,n_\lambda}$, s the experimental (environmental or system) parameters $(\mathrm{s}_q)_{q=1,\dots,n_\mathrm{s}}$ and z the model outputs $(z_m)_{m=1,\dots,n_z}$.

With a specified acceptance threshold θ, we define the model solution space K as the set of fits, sets of kinetic parameter values in agreement with a given set of experiments with associated experimental parameters S_exp and measurements Y:

$$\mathrm{K}_{\mathrm{M},\mathrm{S}_\mathrm{exp},Y} := \{\lambda : \Delta(\mathrm{M}(\lambda, \mathrm{S}_\mathrm{exp}), Y) < \theta\} \tag{S.3}$$

where Δ is an error or distance metric for corresponding model outputs and experimental data, for instance a mean squared (absolute) logarithmic error (Eq. S.8).

From this model solution space, a finite number of ordered kinetic parameter sets λ with increasing error is described as fit ensemble (FE):

$$\mathrm{FE} = (\mathrm{FE}_l)_{l=1,\dots,n_\mathrm{FE}} : \mathrm{FE}_l \in \mathrm{K} \wedge (\Delta(\mathrm{M}(\mathrm{FE}_l, \mathrm{S}_\mathrm{exp}), Y) < \Delta(\mathrm{M}(\mathrm{FE}_{l+1}, \mathrm{S}_\mathrm{exp}), Y)) \tag{S.4}$$

We define the sequence of outputs of a model M for all kinetic parameter sets of a FE of size n_{FE} in association with a single set of experimental parameters s as ensemble solution (ENS):

$$\mathrm{ENS} = (\mathrm{M}(\mathrm{FE}_l, \mathrm{s}))_{l=1,\ldots,n_{\mathrm{FE}}} \tag{S.5}$$

For the evaluation of the NC method, collections of ensemble solutions with varying experimental parameters are generated, a single ensemble solution is used to obtain an ensemble spread or a parameter constraint potential.

Supplementary Note 2: Equations for ensemble mean and standard deviation

Ensemble mean and ensemble standard variation for the determination of the ensemble spread are defined as:

$$\overline{Z_m} = \frac{\sum_{l=1}^{n_{\mathrm{FE}}} Z_{lm}}{n_{\mathrm{FE}}} \tag{S.6}$$

$$\sigma_m = \sqrt{\frac{1}{n_{\mathrm{FE}} - 1} \sum_{l=1}^{n_{\mathrm{FE}}} (Z_{lm} - \overline{Z_m})^2} \tag{S.7}$$

where n_{FE} is the number of fits in the fit ensemble and outputs in the associated ensemble solution, and Z_{lm} the output of the model for fit l in the ensemble and data point m.

Supplementary Note 3: Parameter boundary constraint potential metric with reduced sample density

In order to reduce the computational effort required for the evaluation of the parameter constraint potential metric, we sort ensemble solutions according to their error towards the mean of all predictions, and take into account if the difference of 50 % of $N_{\mathrm{Y},0}$ between the individual prediction and the ensemble solution average is negative or positive. Or simply put, if the fit's associated chemical half-life is smaller or larger than the average chemical half-life of the ensemble solution. By applying this order of fits on the ensemble solution, the parameter constraint potential can be approximated by evaluating a much smaller, evenly distributed fraction of all predictions as subset-forming elements, as demonstrated in Fig. S1.

Note that the comparison of parameter constraint potentials across various kinetic parameters or fit ensemble is problematic, as the measure is sensitive to the overall distribution of their individual values in the fit ensemble. We suggest to take this into account by subtracting the "background value" of the parameter constraint potential at its minimum in the constraint potential map from all parameter constraint potential values of this parameter. Additionally, the parameter constraint potential can be normalized by dividing the values by the logarithmic difference of the previous upper and lower boundary of the corresponding parameter. Such post-processing steps strongly depend on the distributions of kinetic parameter values in the underlying fit ensemble.

Furthermore, the initial assumption that every fit in the fit ensemble has a similar probability to represent the true physical values only applies, if the acceptance threshold for fit acceptance is selected sufficiently low. In applications where this is not the case, a weight could be associated with each subset, inversely proportional to the subset-forming fit's error in comparison with the previous experiments. This way, the larger probability of better fits in the fit ensemble to represent or resemble the physical truth is taken into account for parameter constraint potential calculation.

Supplementary Note 4: Oleic acid ozonolysis system applied in this study

We select r_p and $[\mathrm{O_3}]_\mathrm{g,0}$ (Tab. 1) as variable system parameters. The third system parameter $[\mathrm{OL}]_\mathrm{b,0}$ is set to 1.89 $\cdot 10^{21}$ cm^{-3} to simplify this exemplary application and avoid the curse of dimensionality. In the range $[1 \cdot 10^{-6},\ 1 \cdot 10^{-2}$ cm$]$ for r_p and $[1 \cdot 10^{10},\ 1 \cdot 10^{16}$ cm$^{-3}]$ for $[\mathrm{O_3}]_\mathrm{g,0}$, we define a 100×100 log-uniform grid of potential experiments. For each point on this grid, we obtain a PE based on a pre-sampled FE with the associated experimental parameters r_p and $[\mathrm{O_3}]_\mathrm{g,0}$. Large values on the associated ES map are considered system parameters for potential experiments that are likely to lead to a large reduction of model solution space. Naturally, we propose the r_p and $[\mathrm{O_3}]_\mathrm{g,0}$ values associated with the absolute ES maximum to be the best experimental set-up to restrict the model solution space. Based on the system parameters, as well as the model outputs, we furthermore reject proposed experiments that would be difficult or impossible to conduct in a laboratory. To be accepted, suggested experiments may not exceed the following boundaries:

- 50 nm < r_p < 100 μm
- 10^{12} cm^{-3} < $[\mathrm{O_3}]_\mathrm{g,0}$ < 10^{16} cm^{-3}
- 1 s < predicted experiment duration < 3 d

As the experiment time can only be derived from the model outputs and is dependent on its kinetic parameters, we compute all simulated experiments with KM-SUB beforehand. All combinations of experimental parameters where at least one set of kinetic parameters leads to a simulated experiment with measurements exceeding the boundaries are not accepted as proposed experiments during the evaluation of the NC. The resulting boundaries are visualized in Fig. S3.

Supplementary Note 5: Surrogate model training

For the generation of the SM, we use feedforward multilayer-perceptrons with a maximum of three hidden layers provided by the Python library Keras (Chollet et al., 2015) and compute 1×10^6 random KM-SUB samples in log-uniform parameter space as training (990,000 samples) and test data (10,000 samples). The individual steps in KM-SUB sampling, data pre-processing, neural network model training and validation are elaborated in detail in Berkemeier et al. (2023). As the required SM is nearly identical with the one presented in this previous work with regards to in- and outputs of the template model, we adapt the suggested hyperparameters and only perform very basic hyperparameter tuning (<10 tested hyperparameter sets) applying 5-fold cross-validation to avoid over-fitting.

The SM selected for further evaluation achieves a test set mean square error (MSE) of 2.46×10^{-3}. The average test set MSE of the five cross-validation models is slightly larger at 3.06×10^{-3} and error variance of the five models low at 1.80×10^{-7}, an indication for no significant over-fitting. Average training time for an individual model on one NVIDIA GeForce GTX 1080 Ti is 6587.0 s (< 2 h). In comparison with the best-performing SM for KM-SUB presented in Berkemeier et al. (2023), we achieved a significant reduction of test errors by focusing brief hyperparameter tuning on the optimization of the individual layers' dropout rates. The hyperparameters of the model selected for this study are: Number of hidden layers: 2, numbers of neurons in layers: (4096, 4096), layer activations: ('relu', 'relu'), layer dropout rates: (0.2, 0.2), learning rate: 0.0001, learning rate decay: no, batch size: 16, epochs: 32.

Supplementary Note 6: Fit ensemble acquisition with KM-SUB and SM

In this study, we use seven experimental data sets of the ozonolysis of oleic acid aerosol available in the literature (Hearn and Smith, 2004; Ziemann, 2005; Gallimore et al., 2017; Müller et al., 2022) and a mean square logarithmic error (MSLE) to quantify the error of a matrix Z of model outputs $Z_{i,j}$ for experiment i with specified experimental parameters, and data point j, in comparison with the matrix Y of the corresponding experimental data $Y_{i,j}$ (Berkemeier et al., 2023):

$$\mathrm{MSLE}(Z,Y) = \frac{\sum_{i=1}^{n_\mathrm{exp}} \frac{\sum_{j=1}^{n_\mathrm{d}} (\log_{10}(Z_{ij}) - \log_{10}(Y_{ij}))^2}{n_\mathrm{d}}}{n_\mathrm{exp}} \tag{S.8}$$

where n_exp is the number of experimental data sets and n_d the number of data points in each set. Note that the measured decomposition steps in the experimental data are not always equal to the default output of the simplified KM-SUB that we use and may require an interpolation. We add an additional data point at $x = 1$, $z = 0$ to all individual model output sequences, as these initial conditions apply in every case (no decomposition at time 0) and apply a second order spline interpolation on the model outputs.

We use the two compared models, KM-SUB and the SM in turn to acquire a fit ensemble of 500 kinetic parameter sets each, using random batch sampling in log-uniform parameter space. Sampled parameter sets which result in a model output with a MSLE falling below θ = 0.0105 are added to the associated fit ensemble. Visualizations of seven ensemble solutions for experimental conditions corresponding to the seven experiments used in this study are shown in Fig. 3. A contrariwise cross-evaluation of each fit ensemble with the opposite model allows an estimation of "false-positive" and "false-negative" errors of the SM, and is visualized in Fig. S4. Plot matrices visualizing the input parameter distributions and densities of both fit ensembles are provided in in Fig. S5 and S6.

Supplementary Note 7: Uncertainty calibration and simulated experiments

For the testing of methods that suggest experiments, we perform simulations that include the generation of artificial experimental results based on KM-SUB assuming a single kinetic parameter set from the fit ensemble as the simulated physical truth. We

add uncertainty to the synthetic data in the form of uncertainty in experimental input variables and output values, mimicking the effect of errors in experimental setup and measurement, respectively. To model experimental uncertainty, we sample the value for each variable experimental parameter of the model and each output from a normal distribution in logarithmic space with the original value as the distribution's mean and a defined Σ_{unc} as its standard deviation. Individual values of Σ_{unc} are used for each experimental parameter (Σ_{rad} and Σ_{O_3}) and one for the outputs Σ_{out}. We define $\text{UC}(\text{I}, \Sigma_{\text{unc}})$ as the function that maps a single or multiple input values I to their uncertainty-values under consideration of the corresponding uncertainty parameter Σ_{unc}.

Σ_{rad}, Σ_{O_3} and Σ_{out} are calibrated with a method that tracks error development throughout multiple random simulated experiments. First, a single kinetic parameter set FE_l is selected and its error in association with the real experimental data Y obtained:

$$\delta_{\text{exp},l} = \text{MSLE}(\text{M}(\text{FE}_l, \text{S}_{\text{exp}}), Y) \tag{S.9}$$

For a matrix of n_{sim} randomly selected experimental parameter sets S_{sim}, under consideration of the boundaries and conditions presented in Suppl. Note 4, and for n_r repetitions, we obtain the following errors:

$$\delta_{\text{sim},l,i} = \frac{\sum_{u=1}^{n_r} \text{MSLE}(\text{M}(\text{FE}_l, \text{S}_{\text{sim},i}), \text{UC}(\text{M}(\text{FE}_l, \text{UC}(\text{S}_{\text{sim},i}, \Sigma_{\text{rad}}, \Sigma_{\text{X}}), \Sigma_{\text{out}})}{n_r} \tag{S.10}$$

where i is the index of an individual experimental parameter set $(\text{s}_q)_{q=1,\ldots,n_{\text{s}}}$ in S_{sim} and l the index of the kinetic parameter set initially selected. Simply put, we quantify the average error between an unmodified model output and multiple artificial experimental outputs with a set of pre-selected Σ_{unc} based on a single set of kinetic parameters from the fit ensemble. For visualization purposes, we apply an arbitrarily order to the simulated experiments and calculate the average error of all - real and simulated - experiments at each iteration v of the simulation:

$$\delta_{l,v} = \frac{\delta_{\text{exp},l} * n_{\text{exp}} + \sum_{i=1}^{v} \delta_{\text{sim},l,i}}{n_{\text{exp}} + n_{\text{sim}}} \tag{S.11}$$

The sequence $(\delta_{l,v})_{v=1,\ldots,n_{\text{sim}}}$ represents the hypothetical error development of multiple experiment simulations where only the simulated uncertainty Σ_{unc} contributes to the error, not the uncertainty of the model mechanism or the kinetic parameter set. Since these errors do contribute to the initial error $\delta_{\text{exp},l}$ to an unknown extent, we suggest a combination of Σ_{unc} that leads to a minor error decrease for kinetic parameter sets with a low $\delta_{\text{exp},l}$ and a larger decrease for those with a large $\delta_{\text{exp},l}$. Three examples are presented in Fig. S10, including the combination of Σ_{unc} that has been selected for this study (Σ_{rad} = 0.05; Σ_{O_3} = 0.02; Σ_{out} = 0.07; panel B). Note that only the concerted effect of the three uncertainty parameters can be calibrated with this approach. Based on individual experimental methods, and associated limitations in accuracy, we only consider combinations for $\Sigma_{\text{out}} > \Sigma_{\text{rad}} > \Sigma_{\text{O}_3}$.

Supplementary Note 8: Sensitivity analysis

To compare the proposed methods with a baseline strategy of experiment selection, we derive sensitivity maps based on the kinetic parameters in the fit ensembles after each simulated experiment, following an intuitive approach of testing conditions where kinetic model parameters are most sensitive. To obtain a total sensitivity map for all kinetic parameters, we vary parameters individually and sum up the absolute model residuals. In detail, we apply the normalized sensitivity models M_{SP} (partial sensitivity) or M_{ST} (total sensitivity) to generate ensemble solutions for the grid of experimental conditions and KM-SUB fit ensemble:

$$M_{SP}(\lambda,s,p) = \left| \frac{\frac{M(\lambda,s)-M(\lambda_{\bar{p}},s)}{M(\lambda,s)}}{\frac{\lambda_p-\lambda_{\bar{p},p}}{\lambda_p}} \right| \tag{S.12}$$

$$M_{ST}(\lambda,s) = \sum_{p=1}^{n_\lambda} \frac{M_{SP}(\lambda,s,p)}{n_\lambda} \tag{S.13}$$

where $\lambda_{\bar{p}}$ are parameter sets with parameter λ_p varied: $\lambda_{\bar{p},p} = \lambda_p * 1.2$. The ensemble solutions are then evaluated in the existing framework of the numerical compass with a simple constraint potential metric that selects the maximum of the average normalized sensitivities of associated experimental conditions.

Supplementary Note 9: Computational effort

The application of the NC method requires evaluations of the applied model in two consecutive steps, fit ensemble acquisition and ensemble solution generation. The application of SM in the fit ensemble acquisition step has been demonstrated in Berkemeier et al. (2023). SM accuracy is dependent on the training data size and has been tested for a wide range of such in Berkemeier et al. (2023). In this study, we arbitrarily selected 1×10^6 as the number of samples in the SM training data, but expect similar results from a SM trained on fewer, e.g., 1×10^5 samples, which scored a nearly identical accuracy (Berkemeier et al., 2023). The overall computational effort associated with the NC method also strongly depends on the choice of dimensions and resolution of the constraint potential map, which makes general statements regarding SM-acceleration difficult. The following numbers are derived from the exemplary application showcased in this manuscript:

Fit acquisition

To obtain 500 fits, the SM sampled 5.71×10^6 kinetic parameter sets in 86,908 s ($\sim$ 1 d) of CPU-time, KM-SUB sampled 2.53×10^6 kinetic parameter sets in $\sim$ 600 d of total CPU-time, distributed onto many CPU on a computer cluster.

Table S1. Simulation argument descriptions and selected values for this study.

Function argument	Selected value	Description
gridsize	[100, 100]	Number of individual values for $[X]_{g,0}$ and r_p on simulated grid
rem_ground_truth	No	If parameter set selected as ground truth is removed from fit ensemble
ignore_ES_frame	1	Minimum distance of simulated experiments from edges of grid
Σ_{rad}	0.05	Simulated experimental uncertainty for r_p [log_{10}(cm)] [1]
Σ_X	0.02	Simulated experimental uncertainty for $[O_3]_{g,0}$ [$log_{10}(cm^{-3})$] [1]
Σ_{out}	0.07	Simulated experimental uncertainty for measurements [log_{10}(s)] [1]
filter_theshold	0.0105	Remove fits from fit ensemble above this acceptance threshold (MSLE, Eq. S.8)
exp_distance	0.2	Minimal distance between two experiments on log_{10} plane for $[O_3]_{g,0}$ and r_p
restrict_exp_duration	[1s, 3d]	Minimal and maximal KM-SUB-predicted duration of experiment to be selected
revive_fits	Yes	If removed fits of the fit ensemble are re-evaluated in later iterations

[1] Defined as standard deviation of normal distribution based on logarithmic original value from which new value is sampled randomly (Suppl. note 7).

Numerical compass method

We apply the NC with a total of 10,000 combinations of experimental parameters (100×100 grid). Each of these 10,000 ensemble solutions contain model predictions for each kinetic parameter set in the fit ensemble (here: 500). The resulting 5×10^6 model evaluations represent a major fraction of the overall computational effort in our workflow. In contrast, 10,000 evaluations of the ensemble spread or parameter constraint potential metrics (one for each ensemble solution) fall within feasibility range on a personal computer within few hours of time (when using the reduced sample density for the parameter constraint potential, Suppl. Note 3). The initial CPU time needed for the generation of 10,000 ensemble solutions is roughly a day for the SM, and more than a year for KM-SUB.

Note that the SM used in this study is trained on only 1×10^6 KM-SUB samples, in contrast to 5×10^6 model evaluations necessary to perform the NC method on a grid with the selected size. Given that the computation of the relevant data with the SM is negligible compared with the computation using KM-SUB, we achieve a speed-up by a factor of ~ 5 for the NC using the KM/SM-hybrid approach. If the SM is also applied for fit ensemble acquisition (i.e., a SM-only application), the speed-up increases to a factor of ~ 7.5.

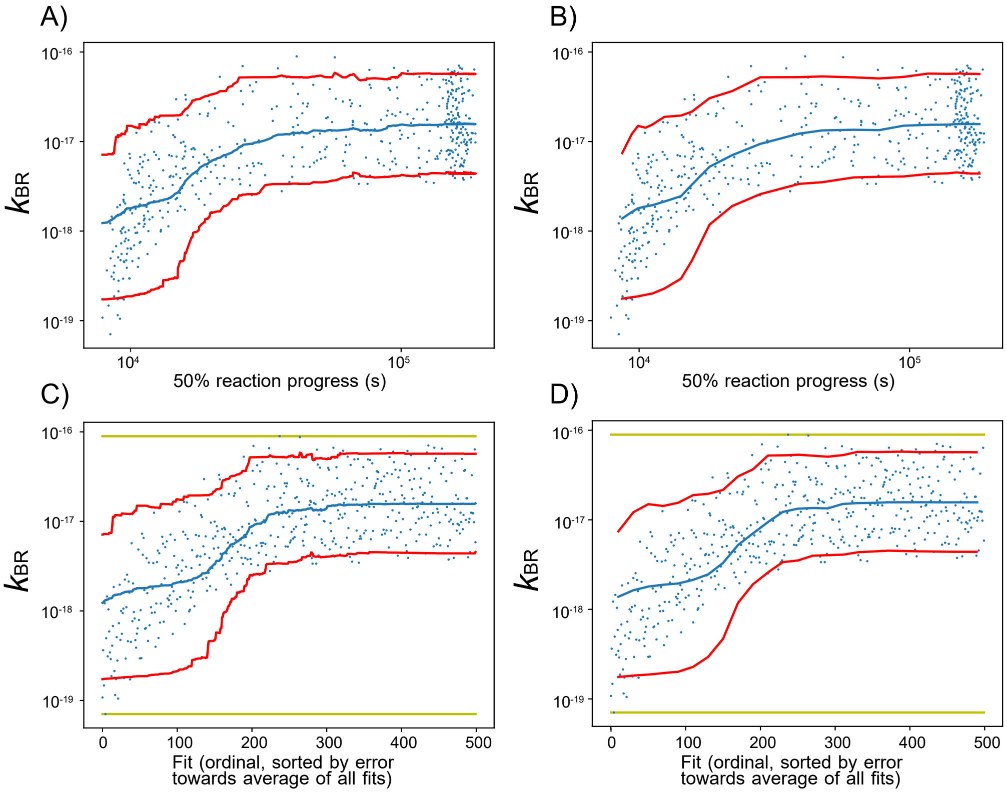

Figure S1. Visualization of the parameter constraint potential metric for the kinetic parameter k_{BR}, KM-SUB, the KM-SUB fit ensemble and the experimental parameters $r_p = 10$ nm, $[O_3]_{g,0} = 4.67 \times 10^{-1}$ ppb and $[OL]_{b,0} = 1.89 \cdot 10^{21}$ cm^{-3}. Panels A and B show the distribution of k_{BR} as a function of chemical half-lives, C and D as a function of an ordinal order of fits according to the MSLE and under consideration, if the chemical half-life is smaller or larger than the one of the ensemble mean. Blue dots represent the values of k_{BR} of individual fits in the sorted fit ensemble. The blue line shows the associated subset average and the red lines the subset 5 and 95 percentiles. The yellow lines represent the absolute minimum and maximum of k_{BR} in the fit ensemble. The parameter constraint potential can be described as the area between the 5-percentile and the minimum plus the area between the 95-percentile and the maximum for the ordinal x-axis (C, D). Panel A and C are based on a fit/subset ratio of 1, panel B and D on a ratio of 20. While the computational effort (with pre-sampled model predictions) to obtain figures B and D is only 5 % in comparison to A and C, only minor differences are visible for the percentiles. Consequently, resulting parameter constraint potentials are almost identical for the two cases, 865.788 for the fit/subset ratio of 1, and 865.793 for the fit/subset ratio of 20.

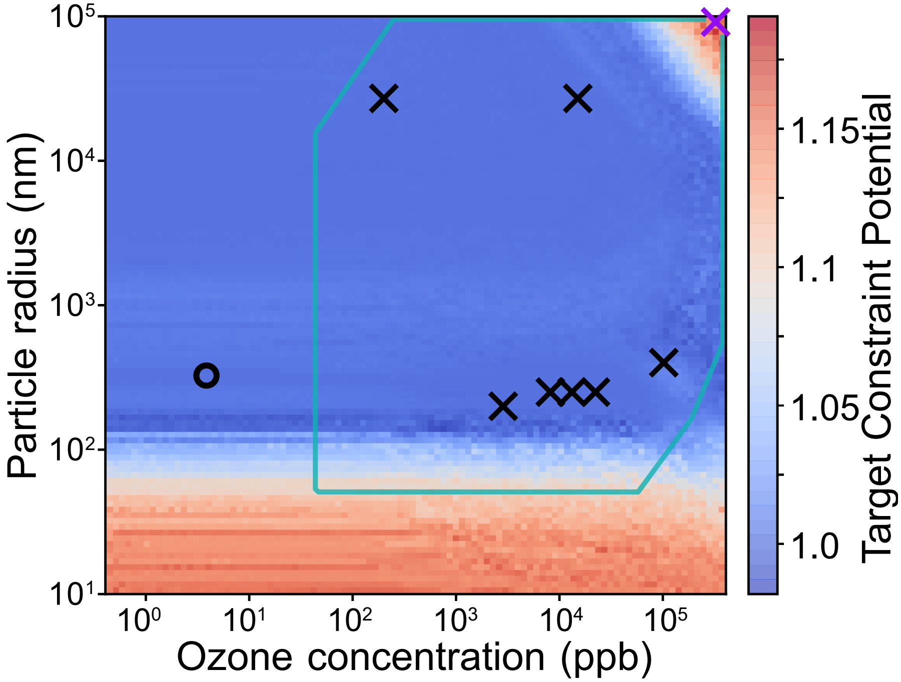

Figure S2. Constraint potential map for the target constraint potential, evaluated by the KM, based on the KM-SUB fit ensemble. The target constraint potential utilizes subsets of hypothetically accepted fits to calculate each subset's ensemble spread at the selected target condition. The target constraint potential is the average ensemble spread at the target condition of all subsets. The selected target in this case represents atmospherically relevant conditions (particle radius: $10^{2.5}$ nm; ozone concentration: 10^{11} cm^{-3}; black circle). Black crosses represent the experimental parameters of the seven real experiments that are used for the initial acquisition of the fit ensemble. The purple cross represents the ensemble spread maximum with satisfied experimental constraint conditions. In basic tests for the oleic acid ozonolysis system, we observed that target constraint potential maps show high similarities to ensemble spread maps if the number of fits in the ensemble is large. Note that this figure was made using the *KineticCompass* module for the Julia programming language.

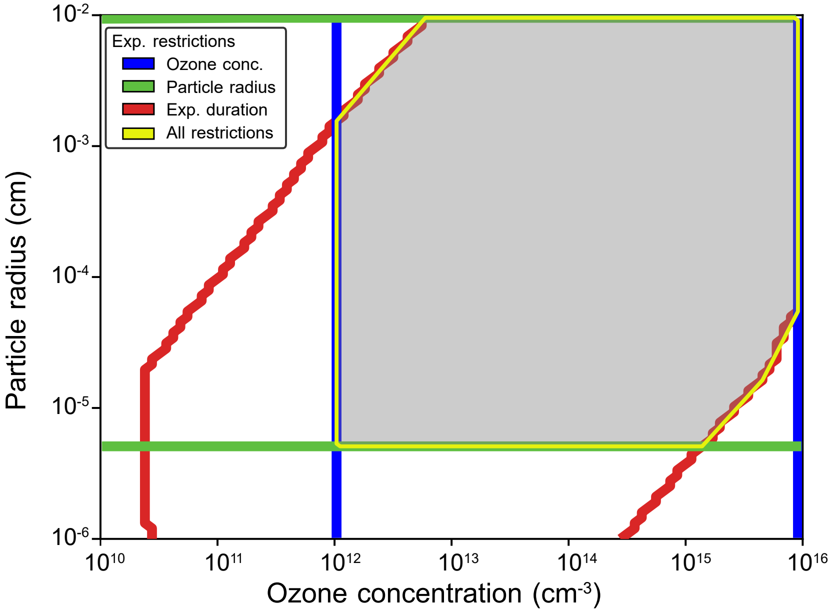

Figure S3. Restrictions for constraint potential maps with regards to experimental feasibility in this study. Blue and green lines show the boundary conditions for the experimental parameters ozone concentration and particle radius, respectively. The red lines frame combinations of parameters where KM-SUB predictions based on all fits in the KM-SUB fit ensemble fall within the required experiment duration (1s < exp_dur < 3d). The yellow box with gray filling shows the area where experiments are accepted for simulation, if proposed by the NC.

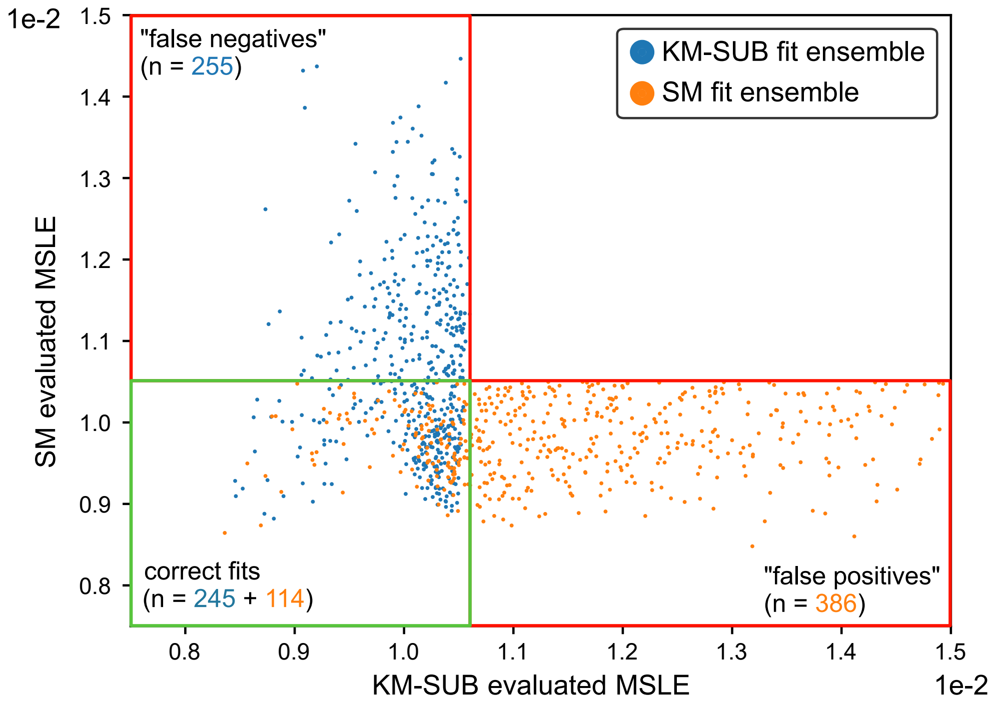

Figure S4. Contrariwise cross-evaluation of the KM-SUB fit ensemble (blue) and the neural network surrogate model (SM) fit ensemble (orange) with regards to mean squared (absolute) logarithmic error (MSLE) in comparison of model outputs with the seven experimental data sets used for initial fit ensemble acquisition. False negative fits in the top left rectangle are KM-SUB fits that are not recognized as fits by the SM. False positive fits in the bottom right rectangle are, in contrary, SM suggested fits with KM-SUB predictions that exceed the associated acceptance threshold $\theta = 0.0105$. In contrast to false positives, false negatives can not be eliminated by re-sampling of the fit ensemble with the KM, and represent a general uncertainty in SM applications (Berkemeier et al., 2023).

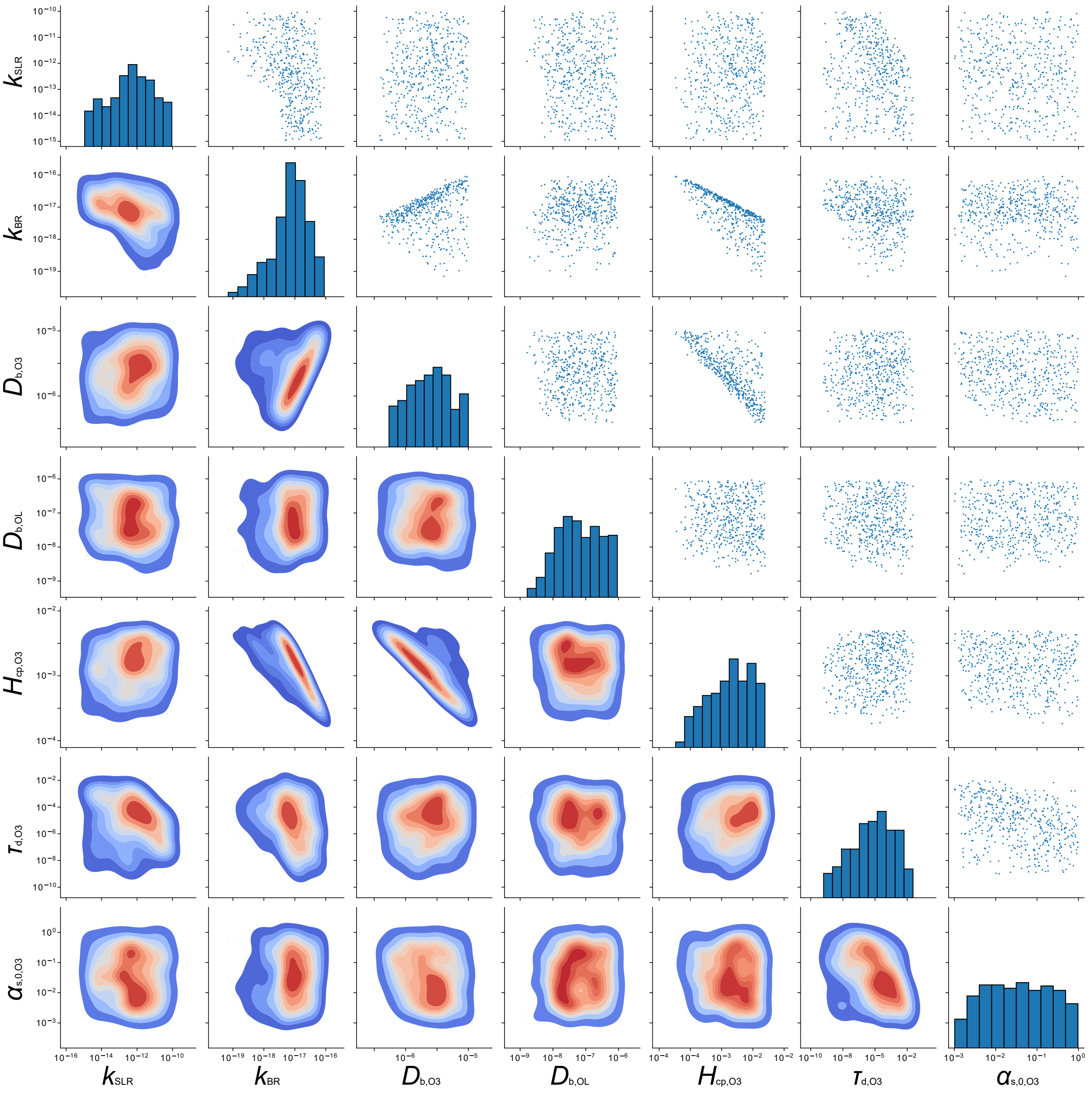

Figure S5. Scatter plot matrix of the KM-SUB fit ensemble (n = 500) with an acceptance threshold θ of 0.0105. The diagonal elements are histograms showing the distributions of the seven kinetic input parameters. The off-diagonal elements are scatter plots (top right) or densities (bottom left) of all combinations of two parameters occurring in the KM-SUB fit ensemble.

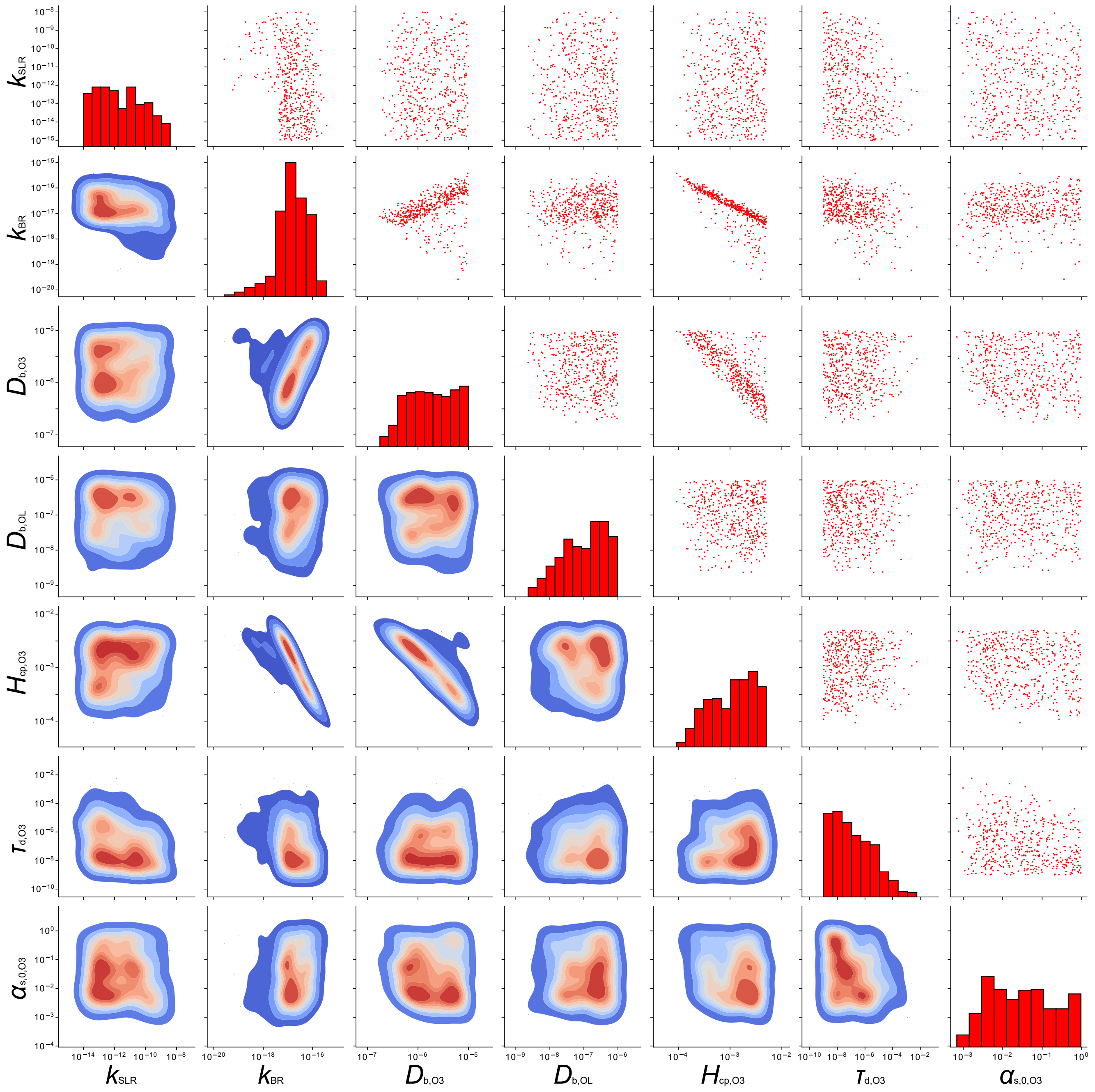

Figure S6. Scatter plot matrix of the SM fit ensemble (n = 500) with an acceptance threshold θ of 0.0105. The diagonal elements are histograms showing the distributions of the seven kinetic input parameters of KM-SUB. The off-diagonal elements are scatter plots (top right) or densities (bottom left) of all combinations of two parameters occurring in the SM fit ensemble.

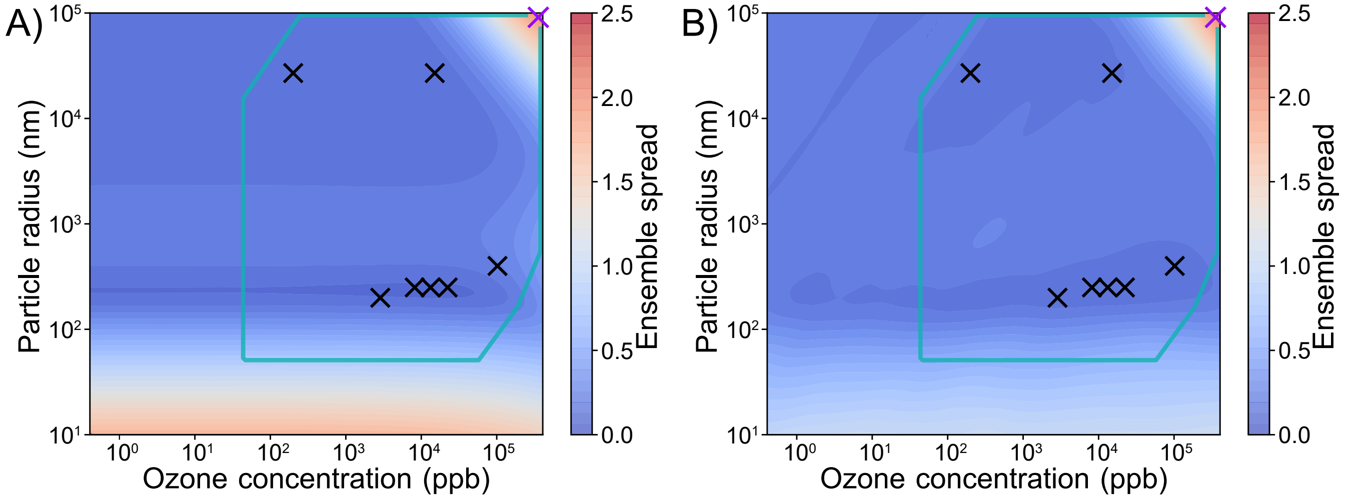

Figure S7. Constraint potential maps for the ensemble spread, evaluated by (A) KM-SUB and (B) SM, based on the KM-SUB fit ensemble and SM fit ensemble, respectively. The teal box frames the area of experimentally accessible conditions with regards to particle radius, ozone concentration and predicted experiment duration (Suppl. Note 4). Black crosses represent the experimental parameters of the seven real experiments that are used for the initial acquisition of the fit ensemble. Purple crosses represent the ensemble spread maximum in each grid with satisfied experimental constraint conditions.

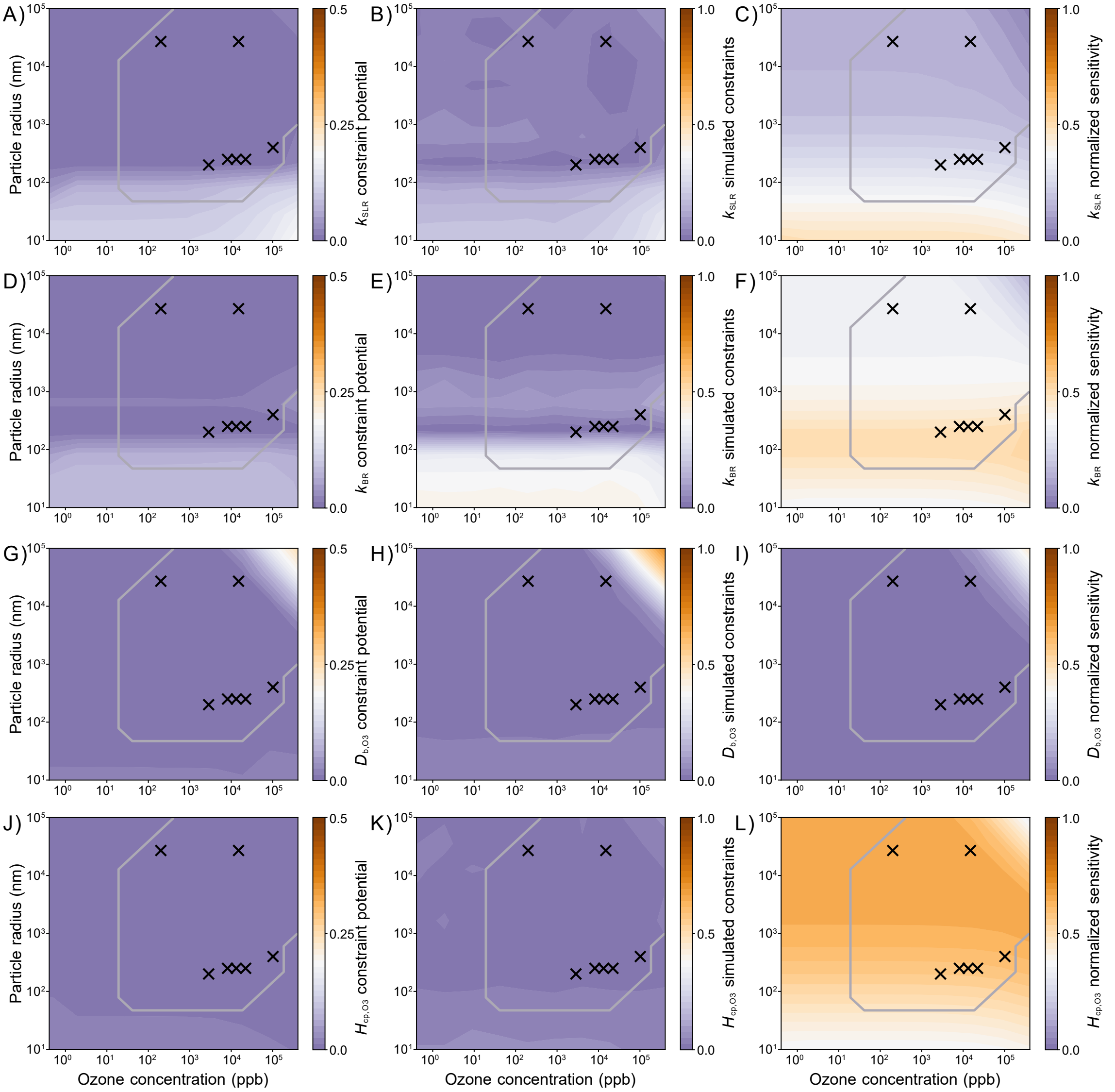

Figure S8. Panels in columns display maps for different methods to select experiments based on approximated constraints for individual parameters: Normalized parameter constraint potential (A, D, G, J), average constraints of 5-95-percentile ranges of parameters in the fit ensemble for brute-force simulation across all fits as simulated truths (B, E, H, K), and normalized partial sensitivities (Eq. S.12) in the KM-SUB fit ensemble (C, F, I, L). Panels in rows show these maps for four kinetic parameters k_{SLR} (A, B, C), k_{BR} (D, E, F), $D_{b,OL}$ (G, H, I), $H_{cp,O3}$ (J, K, L). The gray boxes frame the area of experimentally accessible conditions with regard to particle radius and ozone concentration (Suppl. Note 4). Black crosses represent the experimental parameter sets of the seven real experiments that are used for the initial acquisition of the fit ensemble. The calculations are performed on a reduced 10×10 grid of experimental conditions.

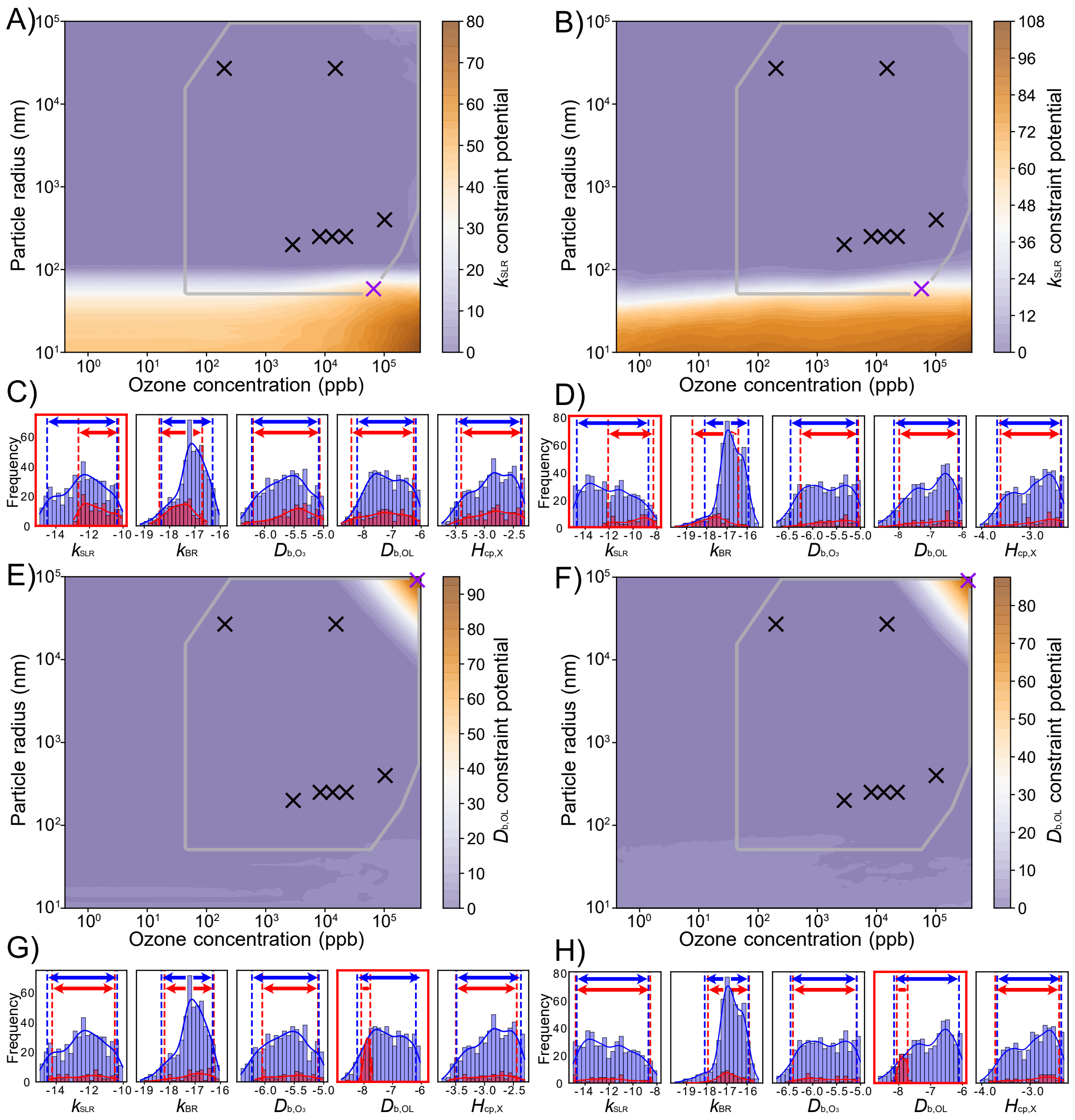

Figure S9. Constraint potential maps for the kinetic parameters k_{SLR} (A, B) and $D_{b,OL}$ (E, F). In (A) and (E), the model KM-SUB is used (KM-only approach), while in (B) and (F), the SM is employed (SM-only approach). The gray box frames the area of experimentally accessible conditions with regard to particle radius and ozone concentration (Suppl. Note 4). Black crosses represent the experimental parameter sets of the seven real experiments that are used for the initial acquisition of the fit ensemble. The purple cross represents the parameter constraint potential maximum with satisfied experimental constraint conditions. The suggested experimental conditions are used to obtain synthetic experimental data by evaluating KM-SUB for the best fit in the KM-SUB fit ensemble. Frequency distributions are shown for individual kinetic parameters in the KM-SUB fit ensemble (C, G) and SM fit ensemble (D, F), before (blue) and after (red) fit filtering with acceptance threshold $\theta = 0.0105$. Blue and red dotted lines and arrows visualize the 5-95 percentile range of each distribution.

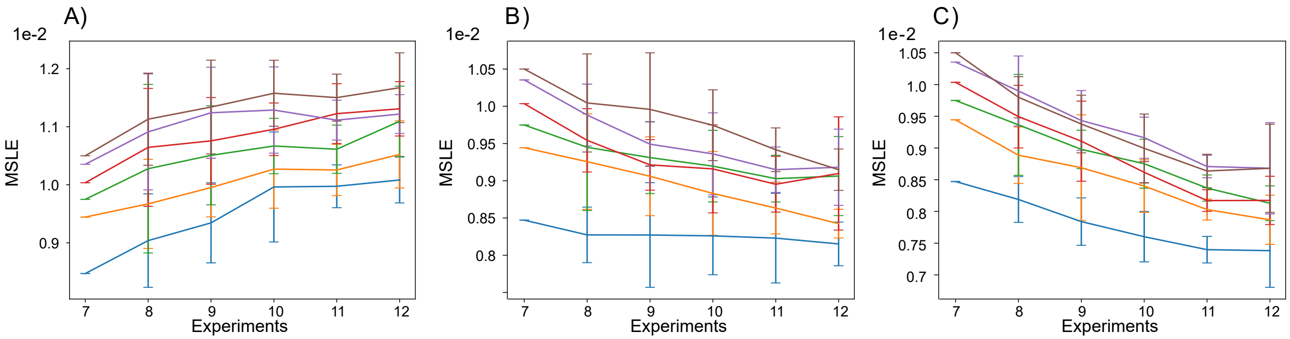

Figure S10. Visualization of the uncertainty calibration method for three sets of uncertainty parameters (Panel A: Σ_{rad} = 0.06; Σ_X = 0.02; Σ_{out} = 0.1; panel B: Σ_{rad} = 0.05; Σ_X = 0.02; Σ_{out} = 0.07; panel C: Σ_{rad} = 0.05; Σ_X = 0.03; Σ_{out} = 0.06) and six selected sets of kinetic parameters that resemble the original $\delta_{\mathrm{exp},l}$ distribution in the fit ensemble. The first data point (7) represents the original $\delta_{\mathrm{exp},l}$ for the seven real experiments of each selected kinetic parameter set (Eq. S.9). In the following, mean $\delta_{l,v}$ errors as well as their standard deviations (error bars) for 20 repetitions and five iterations of the uncertainty calibration are displayed (Eq. S.11). While panels A) and C) show "runaway errors", panel B represents the desired slight error decrease for the best fit and large decreases for fits with a larger $\delta_{\mathrm{exp},l}$. We select these values of Σ_{unc} for the entire study.

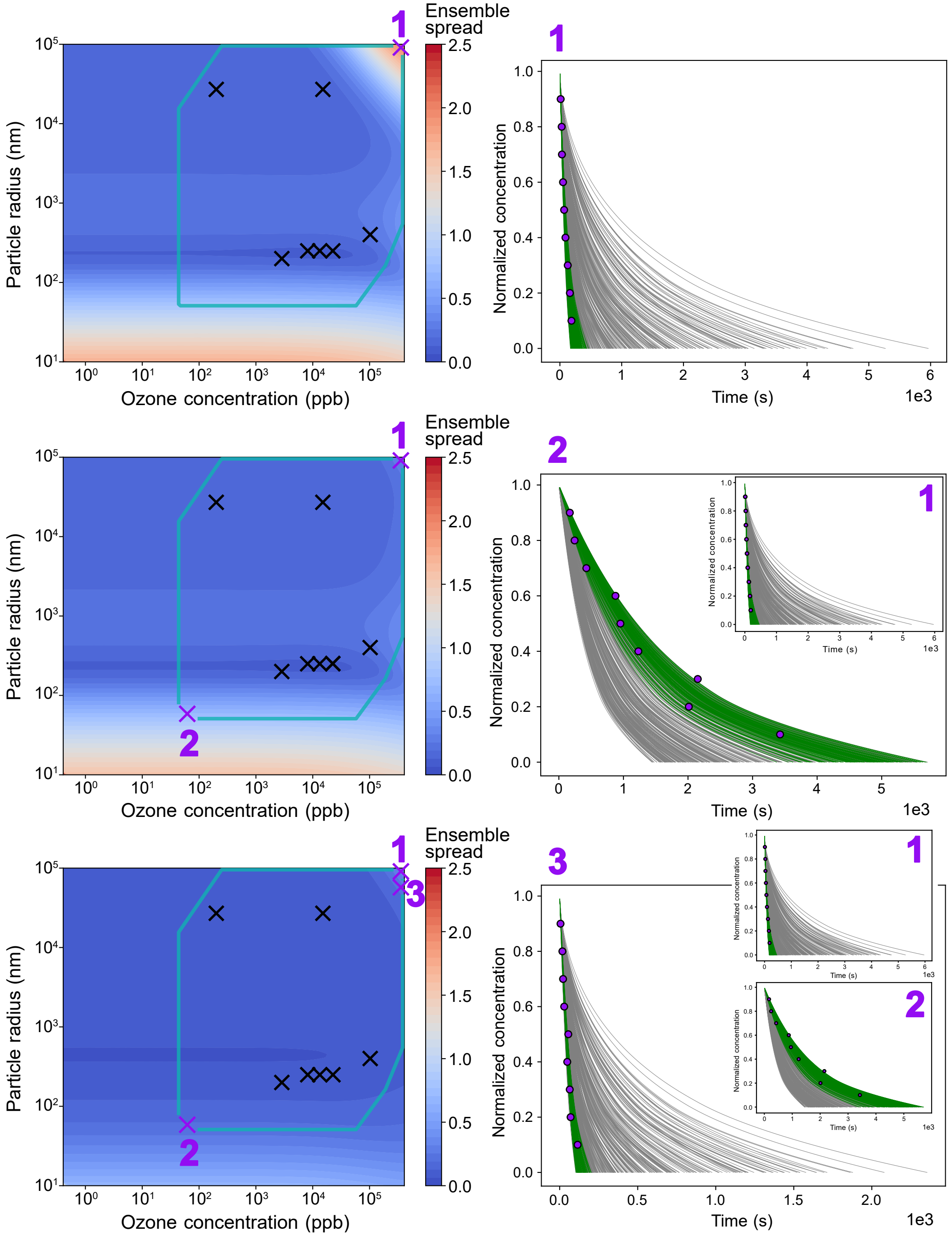

Figure S11. Three iterations of an example simulation for the NC, evaluating the ensemble spread metric with KM-SUB from the KM-SUB fit ensemble. On the left, constraint potential maps for each of three iterations are shown. Plots on the right show ensemble solutions for the selected experimental parameters with the simulated experiment (purple markers), accepted fits (green) and rejected fits (gray) at each iteration. The parameter set selected as simulated truth is the same as in Fig. S13 for the KM/SM-hybrid application. The number of accepted fits in each iteration is 217, 136 and 137, and thus comparably high.

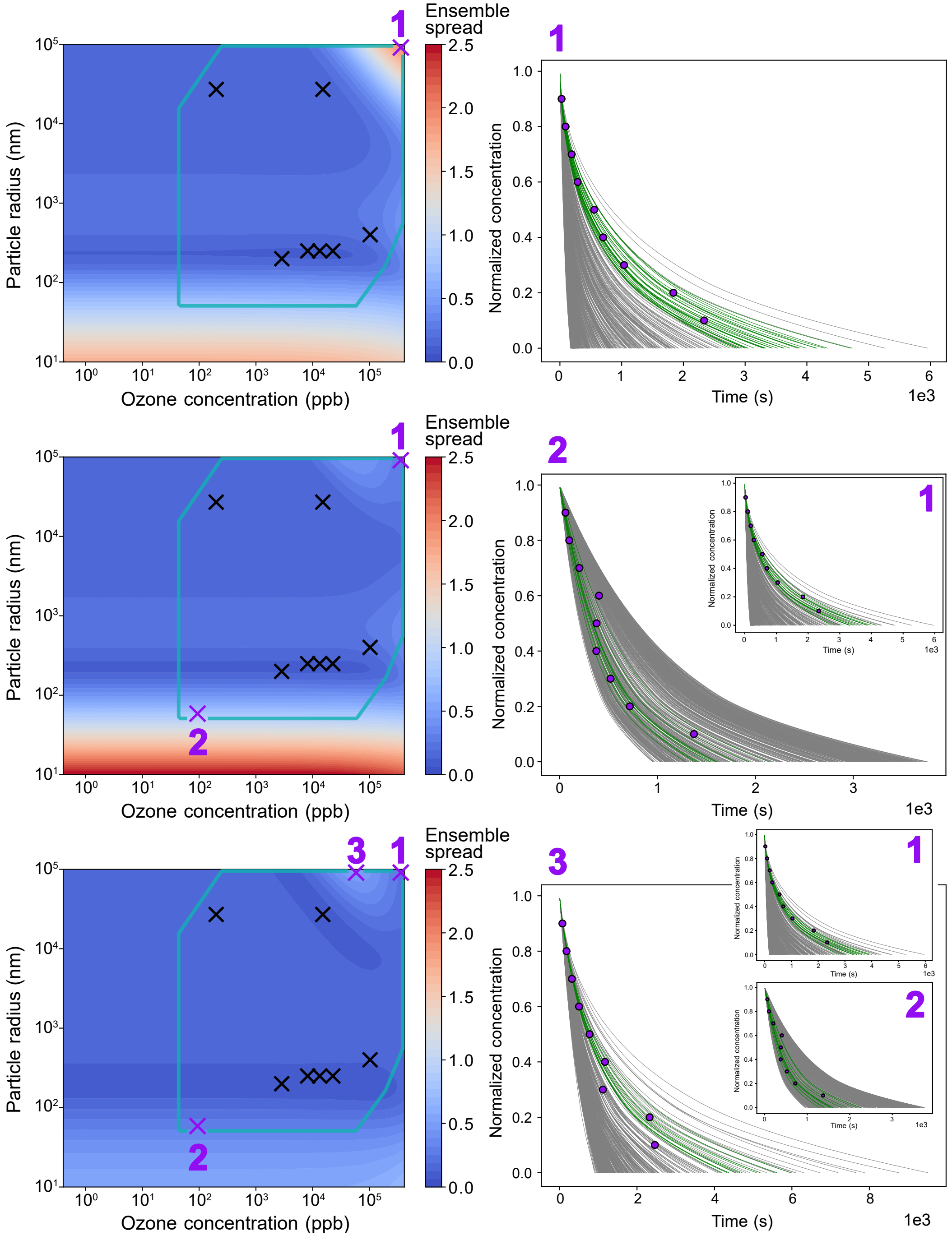

Figure S12. Three iterations of an example simulation for the NC, evaluating the ensemble spread metric with KM-SUB from the KM-SUB fit ensemble. On the left, constraint potential maps for each of three iterations are shown. Plots on the right show ensemble solutions for the selected experimental parameters with the simulated experiment (purple markers), accepted fits (green) and rejected fits (gray) at each iteration. The parameter set selected as simulated truth is the same as in Fig. S14 for the KM/SM-hybrid application. The number of accepted fits in each iteration is 31, 12 and 11, and thus comparably low.

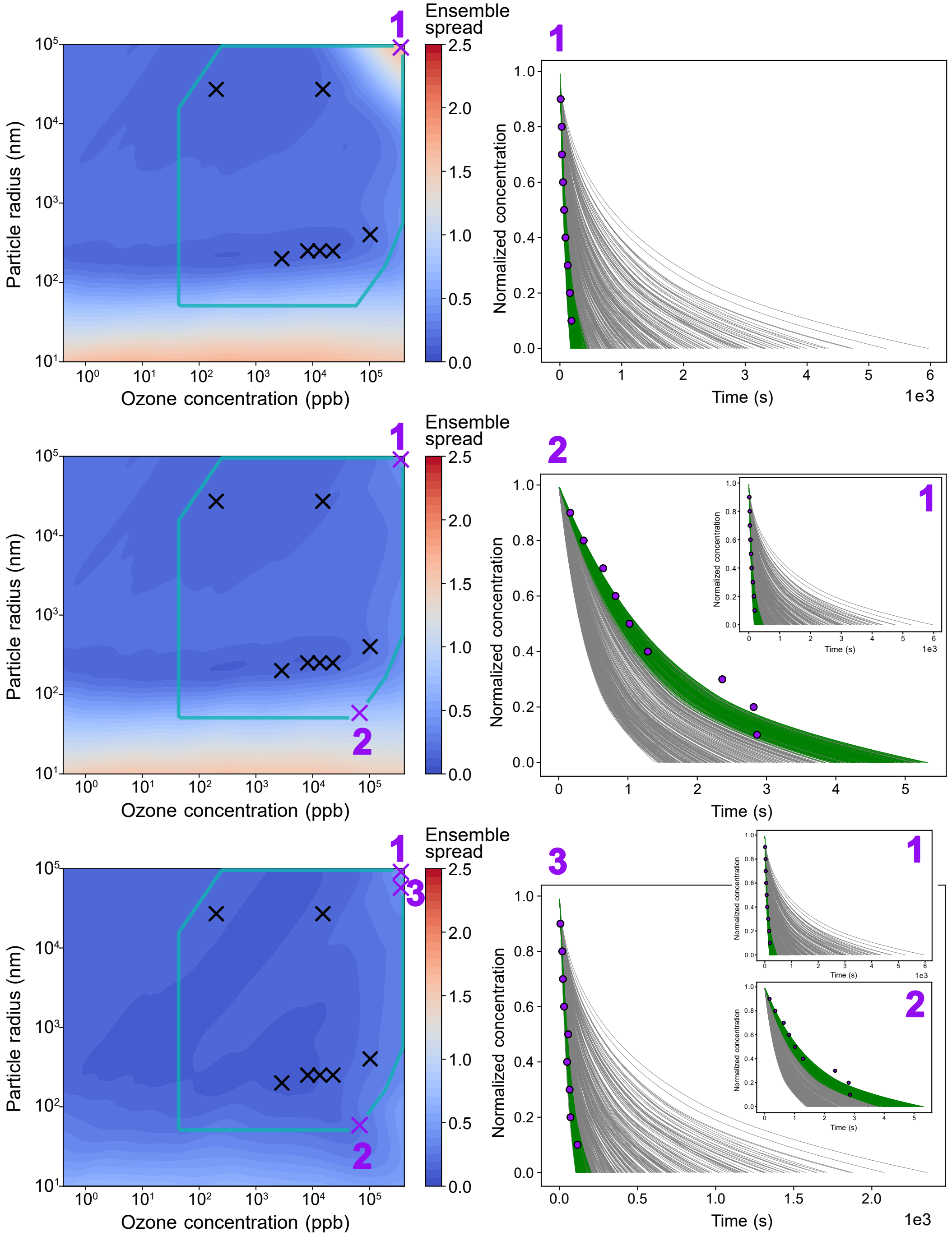

Figure S13. Three iterations of an example simulation for the NC, evaluating the ensemble spread metric with the SM from the KM-SUB fit ensemble. On the left, constraint potential maps for each of three iterations are shown. Plots on the right show ensemble solutions for the selected experimental parameters with the simulated experiment (purple markers), accepted fits (green) and rejected fits (gray) at each iteration. The number of accepted fits in each iteration is 217, 136 and 133, and thus comparably high.

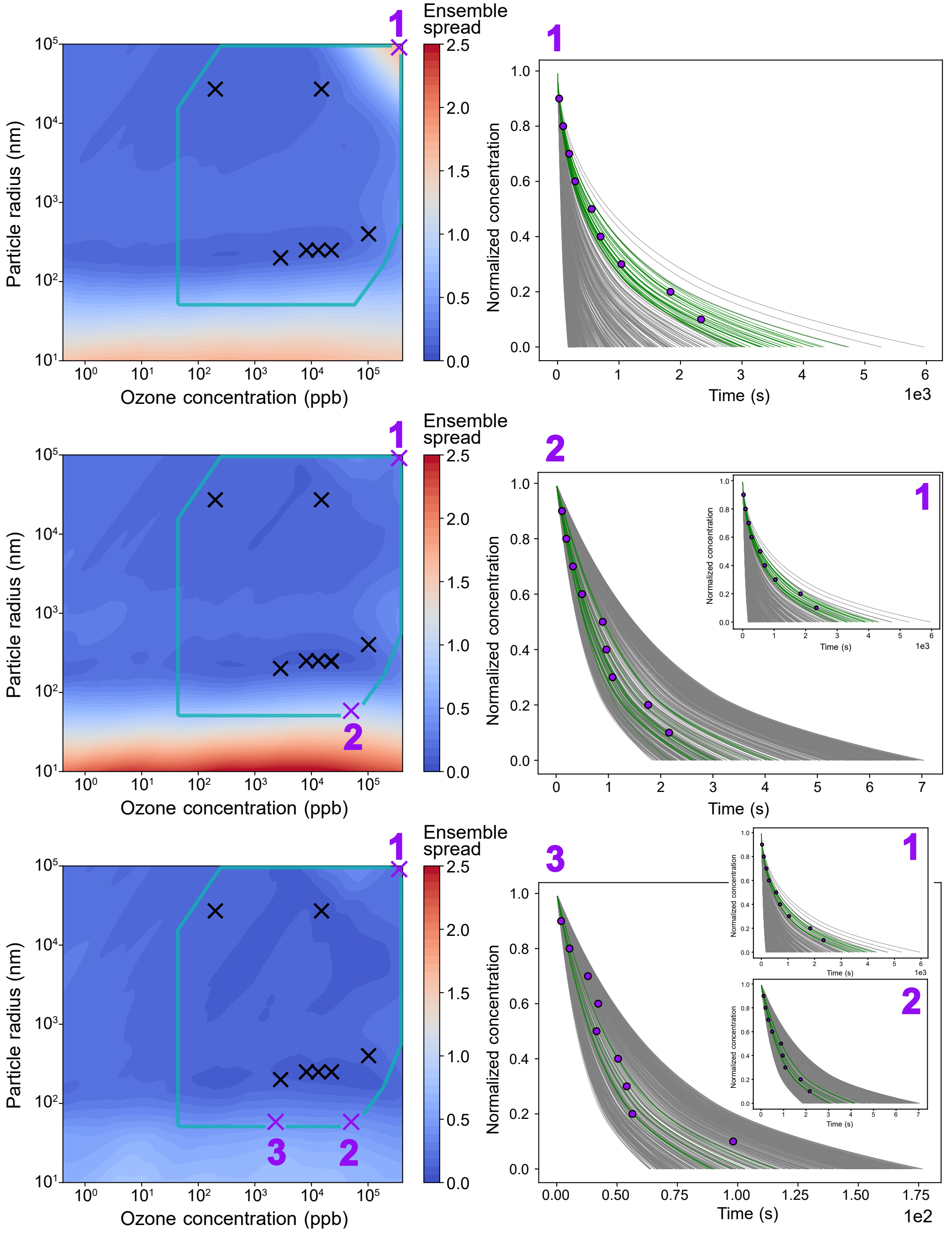

Figure S14. Three iterations of an example simulation for the NC, evaluating the ensemble spread metric with SM from the KM-SUB fit ensemble. On the left, constraint potential maps for each of three iterations are shown. Plots on the right show ensemble solutions for the selected experimental parameters with the simulated experiment (purple markers), accepted fits (green) and rejected fits (gray) at each iteration. The number of accepted fits in each iteration is 31, 15 and 7, and thus comparably low.

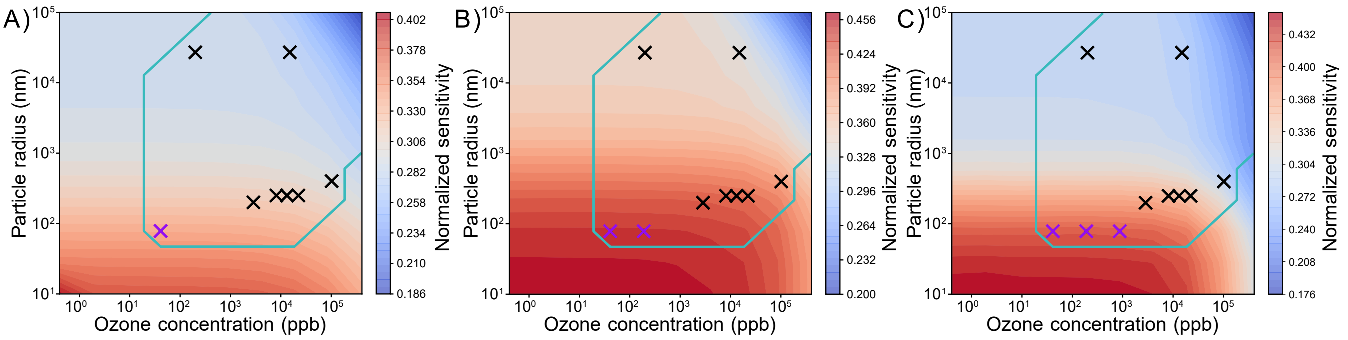

Figure S15. Maps of total sensitivity (Eq. S.13) applied to the fit ensemble during three iterations of an example simulation for the NC, evaluating the normalized parameter sensitivities of KM-SUB using the KM-SUB fit ensemble on a 10×10 grid of experimental conditions. The teal boxes frame the area of experimentally accessible conditions with regards to particle radius, ozone concentration and predicted experiment duration (Suppl. Note 4). Black crosses represent the experimental parameters of the seven real experiments that are used for the initial acquisition of the fit ensemble. The purple crosses represent simulated experiments at the sensitivity maxima with satisfied experimental constraint conditions. The experimental conditions (up to three in panel C) are selected successively in each repetition of the simulation and independent of the simulated truth, synthetic experimental outcome and resulting constraint on the fit ensemble.

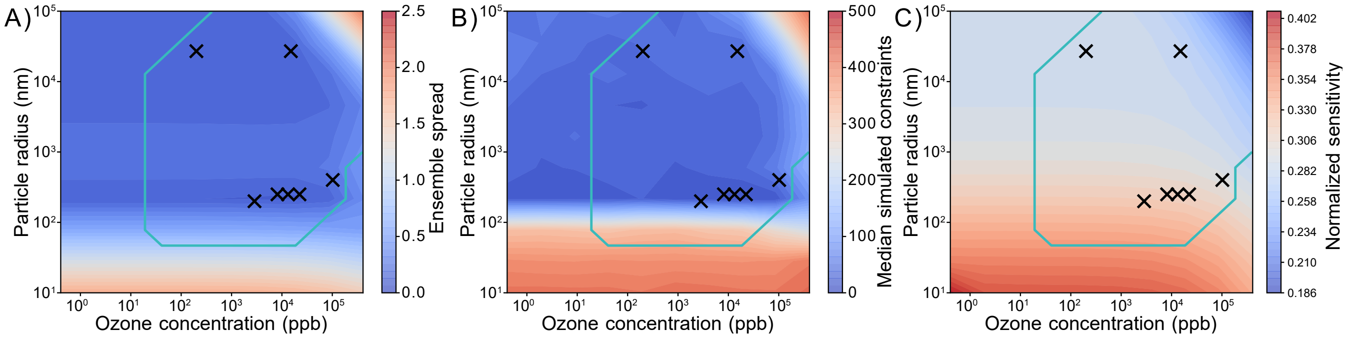

Figure S16. Ensemble spread (KM-SUB; panel A), median brute-force simulated constraints (panel B) and total sensitivities (KM-SUB; panel C) for the KM-SUB fit ensemble in a 10×10 grid of experimental conditions. For the brute force simulation, each fit in the fit ensemble is selected as simulated truth, and the median numbers of fits rejected from the fit ensemble are plotted on the map of experimental conditions in a similar fashion than the constraint potential maps. The map represents the median constraints that can be achieved for the given fit ensemble and assumptions made in the simulation.

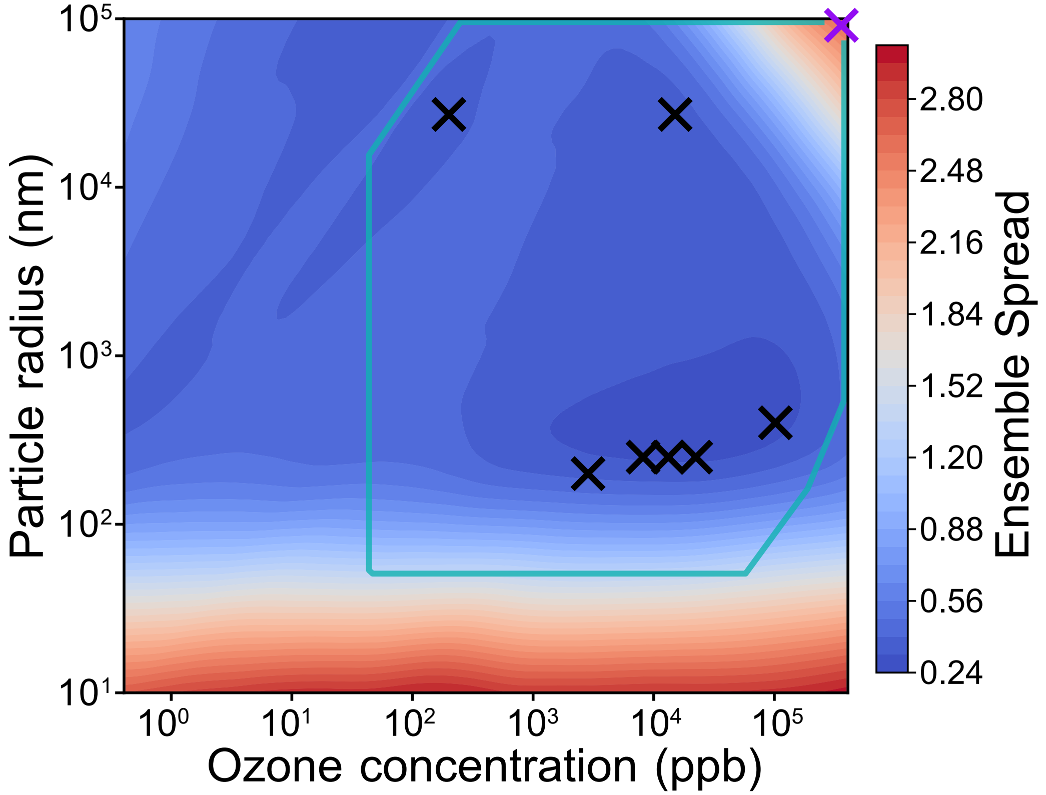

Figure S17. Constraint potential map for the ensemble spread, evaluated by the SM, based on a newly acquired SM fit ensemble with an acceptance threshold of 0.021, a factor two larger than the one used elsewhere in this study. The teal box frames the area of experimentally accessible conditions with regards to particle radius, ozone concentration and predicted experiment duration (Suppl. Note 4). Black crosses represent the experimental parameters of the seven real experiments that are used for the initial acquisition of the fit ensemble. The purple cross represents the ensemble spread maximum with satisfied experimental constraint conditions.

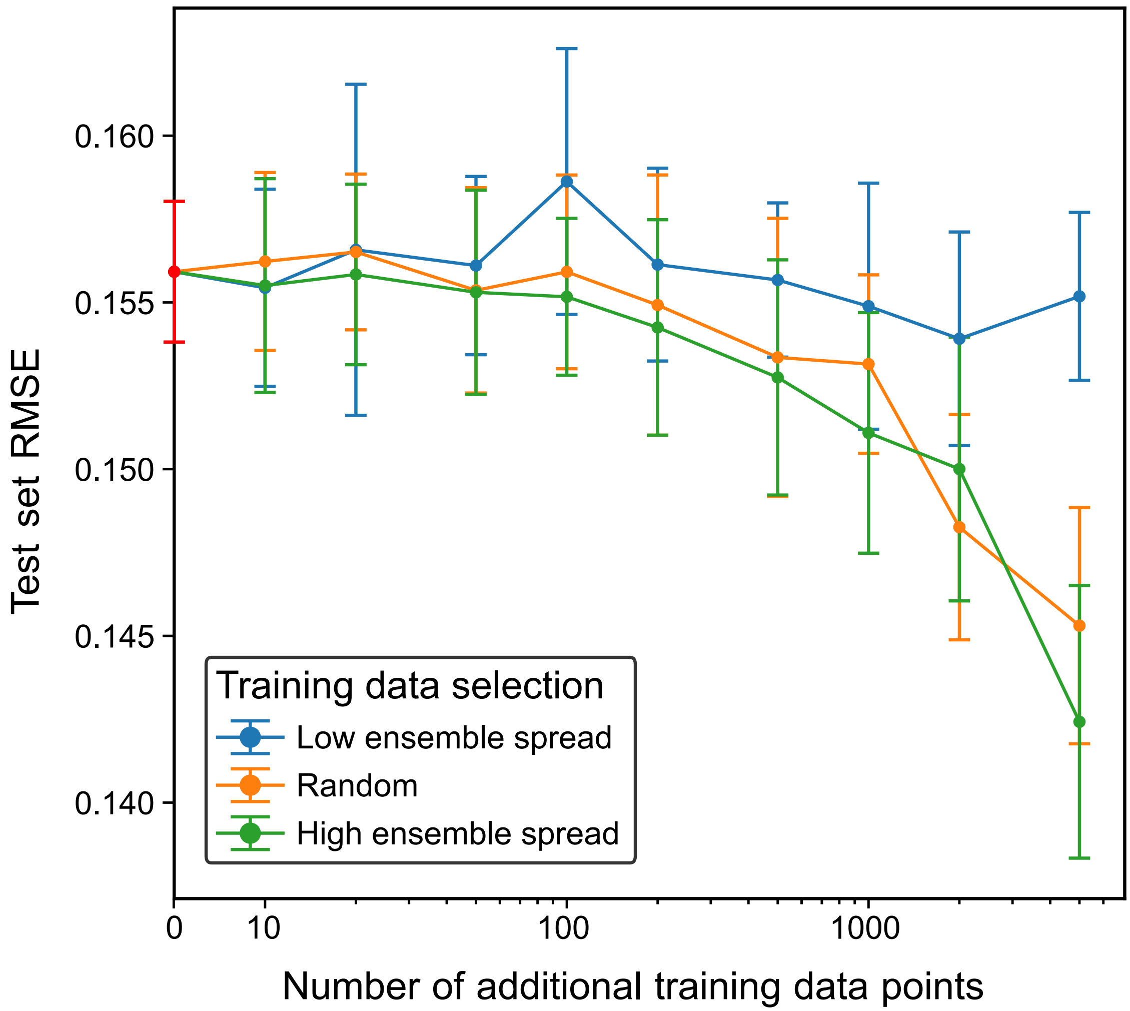

Figure S18. Effect of ensemble spread in additional training data on the QSAR model accuracy of a newly trained model. We train models that parameterize the reduction potential of quinones based on SMILES strings representing molecular structure (Krüger et al., 2022) on a subset of the Tabor_nosulf data (10,000 quinones) (Tabor et al., 2019), using 10-fold cross-validation, and select 1000 quinones as independent test set for all compared models. For the remaining 58,599 quinones in the original data, we determine the ensemble spread using the ensemble predictions of the models from the first step. New models with identical hyper-parameters are then trained on data sets comprised of the original 10,000 quinones plus an additional 10, 20, 50, 100, 200, 500, 1000, 2000 or 5000 quinones from the remaining data. We compare the selection of quinones by largest ensemble spread (green) and lowest ensemble spread (blue) with three different random samples (orange). Markers show mean test set RMSE of the 10 cross-validation models (3×10 for the random selection) in each run, error bars the standard deviation. By addition of molecules with a high ensemble spread, the strongest improvement of the newly trained QSAR models is achieved for almost all data set sizes. However, only a slight difference is observed between the random and high ensemble spread selection.

References

Berkemeier, T., Krüger, M., Feinberg, A., Müller, M., Pöschl, U., and Krieger, U. K.: Accelerating models for multiphase chemical kinetics through machine learning with polynomial chaos expansion and neural networks, Geosci. Model Dev., 16, 2037–2054, https://doi.org/10.5194/gmd-16-2037-2023, 2023.

Chollet, F. et al.: Keras, https://keras.io, 2015.

Gallimore, P. J., Griffiths, P. T., Pope, F. D., Reid, J. P., and Kalberer, M.: Comprehensive modeling study of ozonolysis of oleic acid aerosol based on real-time, online measurements of aerosol composition: Organic Aerosol Model and Measurements, J. Geophys. Res. Atmos., 122, 4364–4377, https://doi.org/10.1002/2016JD026221, 2017.

Hearn, J. D. and Smith, G. D.: Kinetics and Product Studies for Ozonolysis Reactions of Organic Particles Using Aerosol CIMS, J. Phys. Chem. A, 108, 10 019–10 029, https://doi.org/10.1021/jp0404145, 2004.

Krüger, M., Wilson, J., Wietzoreck, M., Bandowe, B. A. M., Lammel, G., Schmidt, B., Pöschl, U., and Berkemeier, T.: Convolutional neural network prediction of molecular properties for aerosol chemistry and health effects, Nat. Sci., 2, e20220 016, https://doi.org/10.1002/ntls.20220016, publisher: John Wiley & Sons, Ltd, 2022.

Müller, M., Mishra, A., Berkemeier, T., Hausammann, E., Peter, T., and Krieger, U. K.: Electrodynamic balance–mass spectrometry reveals impact of oxidant concentration on product composition in the ozonolysis of oleic acid, Phys. Chem. Chem. Phys., 24, 27 086–27 104, https://doi.org/10.1039/D2CP03289A, 2022.

Tabor, D. P., Gómez-Bombarelli, R., Tong, L., Gordon, R. G., Aziz, M. J., and Aspuru-Guzik, A.: Mapping the frontiers of quinone stability in aqueous media: Implications for organic aqueous redox flow batteries, J. Mater. Chem. A, 7, 12 833–12 841, https://doi.org/10.1039/c9ta03219c, 2019.

Ziemann, P. J.: Aerosol products, mechanisms, and kinetics of heterogeneous reactions of ozone with oleic acid in pure and mixed particles, Faraday Discuss., 130, 469, https://doi.org/10.1039/b417502f, 2005.
